# Using the global randomization test as a Mendelian randomization falsification test for the exclusion restriction assumption

**DOI:** 10.1007/s10654-024-01097-6

**Published:** 2024-02-29

**Authors:** Louise A. C. Millard, George Davey Smith, Kate Tilling

**Affiliations:** 1 MRC Integrative Epidemiology Unit (IEU), University of Bristol, Bristol, UK; 2 Population Health Sciences, Bristol Medical School, University of Bristol, Bristol, UK

**Keywords:** Mendelian randomization, Exclusion restriction assumption, Falsification test, Selection bias, Horizontal pleiotropy

## Abstract

Mendelian randomization may give biased causal estimates if the instrument affects the outcome not solely via the exposure of interest (violating the exclusion restriction assumption). We demonstrate use of a global randomization test as a falsification test for the exclusion restriction assumption. Using simulations, we explored the statistical power of the randomization test to detect an association between a genetic instrument and a covariate set due to (a) selection bias or (b) horizontal pleiotropy, compared to three approaches examining associations with individual covariates: (i) Bonferroni correction for the number of covariates, (ii) correction for the effective number of independent covariates, and (iii) an r^2^ permutation-based approach. We conducted proof-of-principle analyses in UK Biobank, using CRP as the exposure and coronary heart disease (CHD) as the outcome. In simulations, power of the randomization test was higher than the other approaches for detecting selection bias when the correlation between the covariates was low (r^2^ < 0.1), and at least as powerful as the other approaches across all simulated horizontal pleiotropy scenarios. In our applied example, we found strong evidence of selection bias using all approaches (e.g., global randomization test *p* < 0.002). We identified 51 of the 58 CRP genetic variants as horizontally pleiotropic, and estimated effects of CRP on CHD attenuated somewhat to the null when excluding these from the genetic risk score (OR = 0.96 [95% CI: 0.92, 1.00] versus 0.97 [95% CI: 0.90, 1.05] per 1-unit higher log CRP levels). The global randomization test can be a useful addition to the MR researcher’s toolkit.

**Supplementary Information:**

The online version contains supplementary material available at 10.1007/s10654-024-01097-6.

## Introduction

Mendelian randomization (MR) is a valuable approach to test for causal effects using observational data, generally using a genetic instrumental variable (IV) to proxy for the exposure of interest [1–4]. However, three core assumptions need to be made, to be able to test for a causal effect using MR, and violations of these assumptions may bias results [5]. These three assumptions are: (1) the IV is associated with the exposure (relevance assumption), (2) there is no unmeasured (i.e., unaccounted for) confounding between the IV and the outcome (independence assumption) and (3) the association of the IV and the outcome is entirely via the exposure (exclusion restriction assumption). To estimate the magnitude of (not just test for) an effect a further assumption of monotonicity or homogeneity is required [4, 6]. The independence assumption may be violated by confounding due to population stratification, by dynastic effects and assortative mating [7]. The exclusion restriction assumption may be violated due to horizontal pleiotropy, where the genetic variant affects the outcome along pathways that are not via the exposure, or linkage disequilibrium. Selection bias can also violate the exclusion restriction assumption by inducing a pathway between the IV and confounders through conditioning on a collider [8].

While only the relevance assumption can be directly tested (by testing the strength of the association of the exposure with the IV), the independence and exclusion restriction assumptions can be investigated with sensitivity analyses and falsification tests that test for evidence that these assumptions do not hold. A common falsification test for the independence assumption is to test for covariate prevalence difference (also known as covariate balance), by testing the association of the IV with a set of potential confounders. Provided these factors are not on the causal path between the IV and exposure, or the exposure and outcome, the IV should not be associated with these factors if the independence assumption holds [9]. For example, *cis* CRP genetic variants were not found to be related to risk factors for cardiovascular disease [10, 11]. Bias can also be estimated, as the covariate prevalence difference divided by the exposure prevalence difference, and displayed in confounding bias plots [9, 12]. This is useful when a researcher wants to compare the potential bias due to confounding in an IV analysis with that of a conventional multivariable regression, as the bias in the causal MR estimate depends also on the strength of the effect of the IV on the exposure [9]. For example, confounding bias plots have been used to assess the potential bias in IV studies of myopia [13] and education [13, 14].

**Fig. 1:**
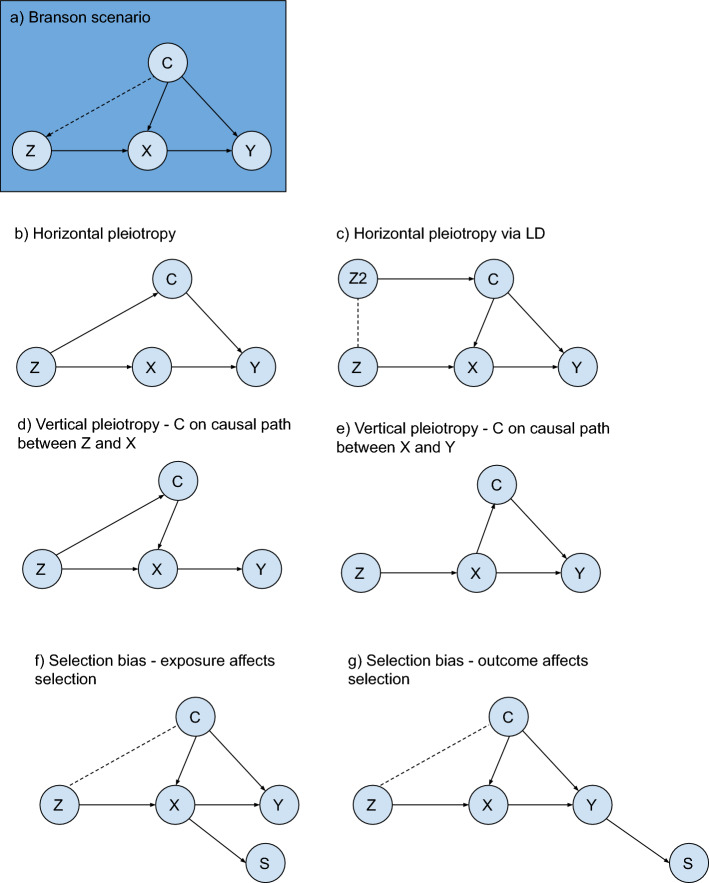
Example scenarios where covariate imbalance may be seen. Z: instrumental variable; X: exposure; Y: outcome; C: covariates; S: selection. Branson scenario (a) assumes covariates C are measured prior to Z and X and may affect both X and Y (dashed arrow indicates association tested using the randomization test). (b–g) Example MR scenarios through which covariate imbalance can occur. In figure (c) dashed line indicates linkage disequilibrium (LD) between genetic variants). Figures (f) and (g) show two example scenarios through which covariates C become correlated with instrument Z due to selection bias [40]. Note that in the case of (g), where selection is determined solely by the outcome, selection bias may be induced when X affects Y; when X does not affect Y selection bias will not be induced [41]

A recent study proposed an approach to compare balance or bias of an IV analysis with what would be expected from a randomized experiment [15]. Given a set of covariates, C, their approach—which we refer to as the global randomization test—uses permutation testing to test whether a binary instrument *Z* is as-if randomized according to *p(Z|C)*, by comparing the observed test statistic (e.g., covariate bias or balance) with that which we would expect if this were true (i.e. no difference in *C* across values of *Z*). They suggest using the Mahalanobis distance as a global measure of balance and bias across the set of covariates tested (shown to correlated well with bias for a binary outcome [16]). Unlike previous IV studies that either reported the Mahalanobis distance directly or the summary percentage change of imbalance for the IV compared with the exposure [17–22], the Branson approach [15] uses permutation testing to estimate a P value reflecting the likelihood the observed level of balance would be observed by chance alone. In their study [15] they assume that *C* are measured before *Z* and *X* are assigned, hence assume that there is no alternate path between the IV and outcome via *C* rather than *X* (i.e. the exclusion restriction assumption holds). However, in an MR setting with a genetic IV, an association between Z and a covariate C may be because of violations of either the independence or the exclusion restriction assumptions (or both) (see Fig. 1). Thus, in MR studies the randomization test has potential to be used as a falsification test for both these assumptions, depending on the MR analysis in question.

In this paper we show how the global randomization test can be useful in MR studies, to identify potential bias due to horizontal pleiotropy or non-random selection. We demonstrate the statistical power in these scenarios using simulations and demonstrate how this approach can be used in practice using proof of principle applications.

## Methods

### Overview of the randomization test procedure

The global randomization test approach as presented in [15] has the following steps:

1. Define set of covariates to test—this depends on the specific scenario (see applied examples).
2. Calculate the test statistic *T*, the Mahalanobis distance (with values $\left[ {0,\infty } \right))$, which is a global measure of balance and bias across all covariates tested.
3. Permute the genetic IV N_p_ times and for each calculate the test statistic t, where N_p_ is specified by the researcher. We use N_p_ = 5000 in our simulations and applied examples below.
4. Calculate the P value as the proportion of permutations with a test statistic *t* at least as strong as *T, i.e.* $\left| {t \ge T} \right|/{\text{N}}_{p}$.

We generalize the approach in [15] (that focused on binary IVs) to continuous, ordinal and binary IVs, as described in the following section.

#### Generalising the Mahalanobis distance to allow continuous, ordered categorical and binary variables

We use the Mahalanobis distance as a global measure of balance defined for an IV with two categories as:

$${\text{MD }} = \sqrt {\left( {\bar{C}_{z = 1} - \bar{C}_{z = 0} } \right)^{T} \left[ {cov\left( C \right)} \right]^{ - 1} \left( {\bar{C}_{z = 1} - \bar{C}_{z = 0} } \right)}$$

where C is a $m \times n$ matrix of *m* participants and *n* covariates, and $\bar{C}_{z = a}$ is a vector containing the mean of the covariates for the subset of participants where z = a.

Since MD is affinely invariant, this is also a global measure of bias (i.e. changing prevalence difference $\bar{C}_{z = 1} - \bar{C}_{z = 0}$ to bias measure $\frac{{\bar{C}_{z = 1} - \bar{C}_{z = 0} }}{{\bar{X}_{z = 1} - \bar{X}_{z = 0} }}$ in the above equation, where X is the exposure, would result in the same MD value).

To generalize to IVs with three categories (i.e., SNP dosages) and continuous IVs (i.e., genetic risk scores), we generalize this equation to:

$${\text{MD }} = \sqrt {meandiff\left( C \right)^{T} \left[ {cov\left( C \right)} \right]^{ - 1} meandiff\left( C \right)}$$

where *meandiff(C)* is a vector of length n, of the mean difference of each covariate per 1 unit higher IV. This assumes a linear relationship between the IV and covariates.

We estimate the mean difference using the correlation between z and each covariate ${\text{C}}_{i}$:

$$meandiff\left( {{\text{C}}_{i} } \right) = {\text{cor}}\left( {{\text{z}},{\text{ C}}_{i} } \right) \times \frac{{SD\left( {{\text{C}}_{i} } \right)}}{SD\left( z \right)}$$

where *cor(A, B)* is the Pearson’s r^2^ between A and B, and SD(X) is the standard deviation of X. This approach also assumes a linear relationship between the IV and covariates but is ~12 times faster than estimating the mean difference using linear regression (see Supplementary section S1 for further details). This is particularly helpful for this work as the randomization test uses permutation testing and we conduct extensive simulations. Note that the MD is invariant to the scale of covariates in C, but not invariant to the scale of Z, and the resultant global randomization test P value is independent of both.

### Simulations

We conduct simulations to explore scenarios where the global randomization test may be useful when testing for horizontal pleiotropy and selection bias. We conduct separate simulations to test for selection bias and horizontal pleiotropy, illustrating how the covariates can be chosen to test each scenario. We report the aims, data‐generating mechanisms, estimands, methods, and performance measures of our simulations (the ADEMP approach) [23].

#### Simulation A: Assessing statistical power to detect potential selection bias

This simulation is based on the situation where a researcher wants to determine whether the GRS relates to covariates that are unlikely to be downstream effects of the GRS, such that an association would indicate possible selection bias. Traits that are downstream effects of a GRS and affect selection cannot be assessed here due to their association with the GRS irrespective of selection bias. Furthermore, it is necessary to assume that the genetic determinants of the covariates causing selection are different to, and uncorrelated with, those of the exposure.

*Aim:* To compare statistical power of the global randomization test compared to alternative tests that test each covariate individually, across different (1) number of covariates that affect selection, (2) number of covariates than do not affect selection, and (3) correlations between covariates.

**Fig. 2:**
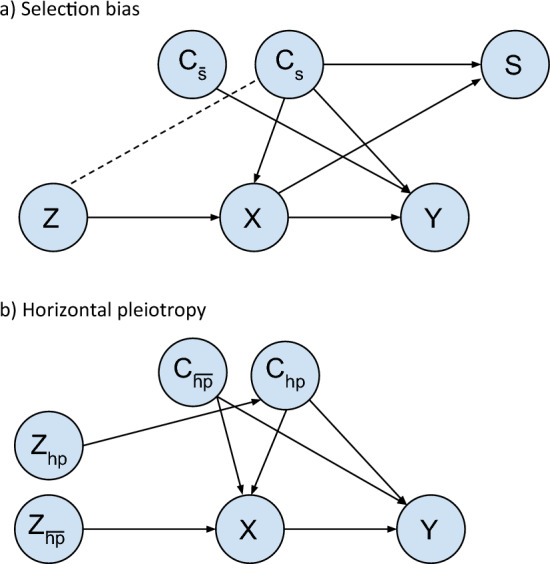
DAGs for simulation data generating mechanisms. **a** Selection bias, **b** Horizontal pleiotropy. DAG (a): Covariates $C_{s}$ and $C_{{\bar{s}}}$ are confounders of X and Y. Covariates $C_{s}$ and exposure X affect selection (S) inducing an association between instrument Z and $C_{s}$*.* X, $C_{s}$ and $C_{{\bar{s}}}$ may affect Y but effects on Y do not impact associations between Z and $C_{s}$ tested by global randomization test. The total effect of the following paths on the DAG is kept constant irrespective of the number of covariates in $C_{s}$ and $C_{{\bar{s}}}$: $C_{s} \to X$; $C_{s} \to Y$; $C_{s} \to S$; $C_{{\bar{s}}} \to X$; $C_{{\bar{s}}} \to Y$*.* Dashed line indicates a statistical association induced through conditioning on S*.* DAG (b): Covariates $C_{hp}$ and $C_{{\bar{hp} }}$ are confounders of X and Y. In this DAG we depict a horizontally pleiotropic instrument that affects Y both via and not via X. While this isn’t necessarily the case (i.e. Z_hp_ might affect Y via X only, or directly (i.e. not via X)) here we are showing an example—the exact relationship between the instruments and X and Y does not impact the randomization test because the randomization test only tests the association between each instrument and the covariate set, and the relationships with X and Y do not impact the strength of these associations (unlike in the selection bias example where e.g. the effect of Z on X impacts the magnitude of the selection induced association between Z and the covariate set).

*Data generating mechanism:* The directed acyclic graph (DAG) on which our data generating mechanism is based is shown in Fig. 2a. We set the proportion selected to 5.5%, based on the UK Biobank recruitment rate where 9.2 million invited and 5.5% of those joined the study. We use a sample size of 920,000, 10% of the number invited in UK Biobank to keep the simulation manageable [24]. A set of covariates C including those affecting selection $C_{s}$ and those not affecting selection $C_{{\bar{s}}}$ are included. We vary the number of covariates affecting selection $N_{cs}$, the number of covariates not affecting selection $N_{{c\bar{s}}}$, the correlation between all variables in C, $r_{c}^{2}$, the variance of X explained by Z, $r_{zx}^{2}$, and the pseudo variance of S explained by C_s_ and X, $r_{cxs}^{2}$. We use a fully factorial design, running our simulation with all combinations of the following values: $N_{cs} \in$ {2, 10, 50}, $N_{{c\bar{s}}} \in$ {2, 10, 50}, $r_{c}^{2}$ $\in$ {0, 0.2, 0.4, 0.8, $N\left( {0,0.1} \right)$}, $r_{zx}^{2} \in \left\{ {0.05, 0.1} \right\}$, and $r_{cxs}^{2} \in \{ 0.05, 0.1, 0.2$}. For the $r_{c}^{2} = N\left( {0,0.1} \right)$ setting the covariate correlations are generated from a normal distribution with mean = 0 and standard deviation (SD) = 0.1, to reflect correlations reported previously [25]. All covariates in C, and exposure X are continuous with mean = 0 and SD = 1. Instrument Z is assumed to be a normally distributed genetic risk score with mean = 0 and SD = 1.

A DAG with simulation parameters is shown in Supplementary Figure 1a. The outcome Y is not modelled as, according to our DAG in Fig. 2a, the association between the SNPs and covariates induced by conditioning on selection does not depend on our definition of Y.

*Estimand or other target:* Our target is the test of the null hypothesis of no association between the GRS and covariate set. Note that in this scenario (in contrast to simulation B, see below) we use the randomization test with the GRS as we assume (see DAG in Fig. 1a) the exposure of interest affects selection, hence selection bias would impact all instruments jointly (and testing using the GRS rather than SNPs individually maximizes statistical power).

*Methods:* We compare the global randomization test with 3 alternative approaches to test the association of the IV with *C*:

- individual tests of each covariate with Bonferroni correction, referred to as test-Bonf.
- individual tests of each covariate with correction for the effective number of tests performed, referred to as test-indep.
- permutation testing where the test statistic is the maximum r^2^ of each of the covariates with the IV, referred to as test-r^2^perm.

To calculate (a) and (b) we first regress the IV on each of the covariates using univariable regression, and find the lowest P value of these results, *pvalue*_*min*_. The test-Bonf P value is then calculated as $\min \left( {1, pvalue_{min} \times {\text{N}}_{c} } \right)$. The P value for test-indep in calculated as ${\text{min}}\left( {1, pvalue_{min} \times {\text{N}}_{I} } \right)$, where *N*_*I*_ is the estimated effective number of independent tests calculated using spectral decomposition [26, 27]. Spectral decomposition estimates the effective number of independent tests from the phenotype correlation matrix, and has been shown to be accurate (compared to the more computationally intensive permutation testing approach) [28].

*Performance measures:* We evaluate statistical power using rejection percentage [23], which is the proportion of simulation repetitions, $n_{sim}$, where the null hypothesis is rejected (see further details in Supplementary section S3). We set $n_{sim}$ = 500 in all our simulations. The four tests (global randomization test, test-Bonf, test-indep and test-r^2^perm) are applied to the same simulated dataset in each simulation repetition.

We repeated these simulations, including only half the covariates in $C_{s}$ and $C_{{\bar{s}}}$ to represent the scenario where only a subset of these covariates is either available or hypothesized to affect selection. We also repeated these simulations in the whole sample (i.e., with no selection) to check that we observe ~5% type-1 error, i.e., around 5% of the permutations incorrectly identify an association between the IV and covariate set.

#### Simulation B: Assessing statistical power to detect potential horizontal pleiotropy

This simulation is based on the situation where a researcher has a GRS but wants to determine whether any of the SNPs included may affect the outcome via horizontally pleiotropic pathways rather than (solely) via the exposure of interest.

*Aim:* To compare statistical power of the global randomization test with alternatives that test the association of a SNP with each covariate individually, to identify whether the SNP acts via a horizontally pleiotropic pathway. We evaluate this across different: (1) numbers of covariates affected and not affected by a horizontally pleiotropic SNP, and (2) magnitude of effect of a horizontally pleiotropic SNP on covariates on the horizontal pleiotropy pathway.

*Data generating mechanisms:* The DAG on which our DGM is based is shown in Fig. 2b. We use a sample size of 500,000 reflecting the size of UK Biobank. We include one horizontally pleiotropic SNP, $Z_{hp} ,$ and one non-horizontally pleiotropic $Z_{{\bar{hp} }}$. We generate a set of covariates C including those affected by $Z_{hp}$, and not affected by $Z_{hp}$, denoted $C_{hp}$ and $C_{{\bar{hp} }}$, respectively. We vary the number of covariates in $C_{hp}$ and $C_{{\bar{hp} }}$, denoted $N_{chp}$ and $N_{{c\bar{hp} }}$, the variance of each covariate in $C_{hp}$ explained by $Z_{hp}$, $r_{zchp}^{2}$, and the correlation between all variables in C, $r_{c}^{2}$. We use a fully factorial design where $N_{chp} \in$ {1, 5}, $N_{{c\bar{hp} }} \in$ {1, 5}, $r_{zchp}^{2} \in$ {0.001, 0.005, 0.01}, and $r_{c}^{2}$ $\in$ {0, 0.2, 0.4, 0.8, ${{N}}\left( {0, 0.1} \right)$}. The exposure X and outcome Y are not modelled as, according to our DAG in Fig. 2b, the association between the SNPs and covariates are not dependent on X or Y.

All covariates in C are continuous with mean = 0 and SD = 1. Each SNP is a 3-category ordinal variable (representing SNP dosages) assuming allele frequencies of 0.8 and 0.2 and assuming Hardy Weinberg Equilibrium (such that P_dosage0_ = 0.64, P_dosage1_ = 0.32, and P_dosage2_ = 0.04). A DAG with simulation parameters is shown in Supplementary Figure 1b.

*Estimand or other target:* Our target is the test of the null hypothesis of no association between each SNP in $Z_{hp}$ and the covariates. Note that in this scenario (in contrast to simulation A, see above) we use the randomization test with the SNPs individually, as we are seeking to identify which SNPs may affect the outcome via horizontally pleiotropic pathways.

*Methods:* As in simulation A, we compare 4 approaches to test the association of the IV with *C*: the global randomization test, test-Bonf, and test-indep and test-r^2^perm.

*Performance measures:* We evaluate statistical power using rejection percentage [23].

We also estimated these performance measures using $Z_{{\bar{hp} }}$ to check that we observe ~5% type-1 error, i.e., around 5% of the permutations incorrectly identify an association between $Z_{{\bar{hp} }}$ and *C*.

### Applied examples

#### Study population

UK Biobank is a prospective cohort of 503 325 men and women in the UK aged between 37 and 73 years (99.5% were between 40 and 69 years). This cohort includes a large and diverse range of data from blood, urine and saliva samples and health and lifestyle questionnaires. UK Biobank received ethical approval from the UK National Health Service’s National Research Ethics Service (ref 11/NW/0382). This research was conducted under UK Biobank application number 16729, using phenotypic dataset ID 48196.

Of the 463,005 UK Biobank participants with genetic data passing quality control [29], we removed 77,758 minimally related participants, 48,233 non-white British participants, and 39 participants who had since withdrawn from the study. Our sample therefore included 336,975 participants. A data flow diagram is provided in Supplementary Figure 2.

#### Example 1: Testing for evidence of selection bias

We assess the potential for selection bias in Mendelian randomization studies in UK Biobank that use C-reactive protein (CRP) as the exposure of interest. A previous GWAS meta-analysis (that did not include UK Biobank) identified 58 SNPs robustly associated with CRP [30]. We generate the CRP GRS as a weighted sum of the 58 SNPs, weighted by the effect size of the CRP-increasing allele of each SNP on CRP.

We use two sets of covariates, a restricted set and a more liberal set. The restricted set comprises just age and sex—two factors that cannot be on the causal path between the (constituent SNPs of the) CRP GRS and outcome. The liberal set were chosen as phenotypes that, given our exposure of interest (CRP) we believe are not likely to either be on the causal pathway between the CRP GRS and CRP, determinants of SNPs in CRP GRS, or downstream effects of CRP (or CRP GRS), such that if an association exists between this set and the CRP GRS we would think it is more likely that this is due to selection bias rather than a causal effect of one or more of the CRP SNPs on (one or more of) these traits. This second set additionally includes socio-economic factors (Townsend deprivation index, age completed full time education), north and east coordinates of home location, and height [31]. Age, sex, home location and education were self-reported at baseline. Sex was validated against genetic sex. Height was measured at baseline, to the nearest cm using a Seca 202 device. The participant’s age when completing full time education was used as a measure of education level. Townsend deprivation index (a score representing the deprivation of the participant’s neighbourhood) was calculated immediately prior to participants joining UK Biobank using their self-reported postcode of residence. This gave 7 variables included in the liberal covariate set.

We ran the global randomization test and alternative approaches to test for an association of the CRP GRS with the restricted and liberal covariate sets, respectively. We also repeated these analyses using the rs2794520 *cis CRP* SNP only, to explore detection of selection bias using the SNP set (in this case just one SNP) that is unlikely to be horizontally pleiotropic. SNP rs2794520 was used as it is only *cis CRP* SNP among the 58 independent SNPs identified in the GWAS (and used in the GRS above).

#### Example 2: Testing for evidence of horizontal pleiotropy among CRP SNPs

We used the global randomization test to identify CRP-associated SNPs that may have horizontally pleiotropic effects on coronary heart disease (CHD). We formed our covariate set using a previous study [10] that found little evidence of an association of *cis* CRP SNPs with a set of CHD risk factors. These risk factors are therefore unlikely to be on the causal pathway between CRP levels and CHD, such that associations with other CRP-associated SNPs (or a combined CRP GRS) would be most likely due to this SNP being horizontally pleiotropic. These risk factors can therefore be used as the covariate set in the randomization test, to test for evidence of horizontal pleiotropy.

Our covariate set comprised the subset of these phenotypes that were measured in the full UKB sample (e.g., some such as LDL cholesterol were only available in the NMR metabolomics UKB subsample), namely: BMI, systolic blood pressure (SBP), diastolic blood pressure (DBP), total cholesterol, HDL cholesterol, apolipoprotein A1, apolipoprotein B, albumin, lipoprotein A, leukocyte count, glucose, smoking pack years, weight and waist hip ratio. Details of the covariates are provided in Supplementary section S4. CHD events were ascertained using both self-reported data and linkage to mortality data and hospital inpatient records (see Supplementary section S5 for further details).

We estimated the causal effect of CRP on CHD using two-stage IV probit regression, first using all CRP SNPs and then using only those not identified as horizontally pleiotropic, using a nominal threshold of *p* < 0.05. We use log transformed CRP levels (mg/L) such that the IV probit regression estimate is the difference in probit index per 1 unit higher log CRP levels. We then took the exponent of 1.6 times the probit regression estimates, to approximate the association in terms of the change of odds per 1 unit higher log CRP levels [32]. We repeated analyses using a threshold of *p* < 0.001, to assess the sensitivity of results to the stringency of SNP selection.

Analyses were performed in R version 4.0.3, Stata version 15 and Matlab r2015a, and all of our analysis code are available at https://github.com/MRCIEU/MR-randomization-test. Git tag v0.2 corresponds to the version of the analyses presented here.

## Results

### Simulation results

#### Using the randomization test to detect selection bias

**Fig. 3:**
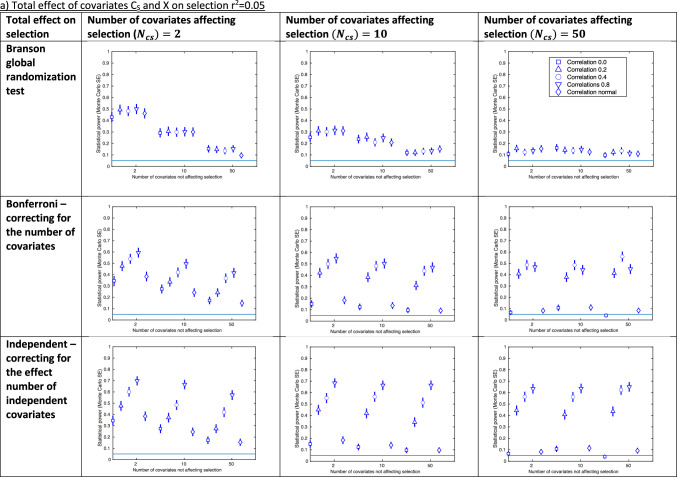

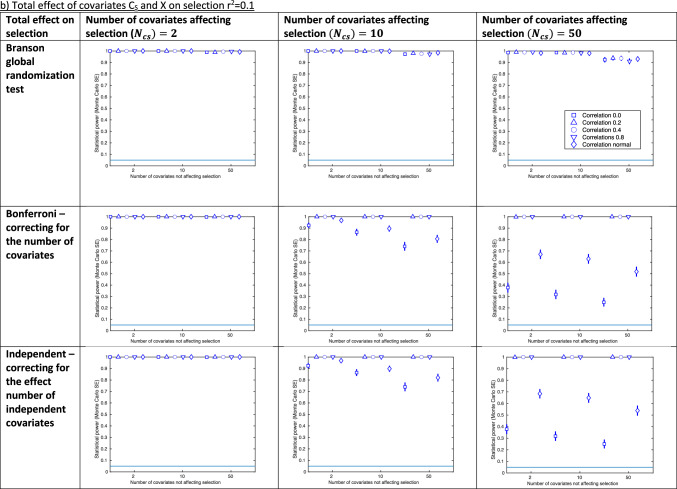
Results of selection bias simulations for instrument strength r^2^ = 0.05 and all covariates included in test **a** Total effect of covariates C_S_ and X on selection r^2^ = 0.05 **b** Total effect of covariates C_S_ and X on selection r^2^ = 0.1. SE: standard error. Total effect on selection: the total effect of covariates C_S_ and X on selection S. Confidence intervals are ± 1.96*MCSE (Monte Carlo standard error). Each graph shows the statistical power of a given approach to identifying covariate imbalance due to selection bias, as the number of covariates not affecting selection (x-axis) and the correlation between covariates (shown in legend) are varied. Each graph shows the results for a particular number of covariates affecting selection* (*${\varvec{N}}_{{{\varvec{cs}}}}$*;* columns), and approach (rows), for a given total effect of covariates C_S_ and X on selection (r^2^ = 0.05 in (a) and r^2^ = 0.1 in (b)). When r^2^ = 0.2 for the total effect on selection, power was at or near 1 in all scenarios. Results for the r.^2^ permutation testing approach were very similar to the independent approach and not shown here (see Supplementary Figure 3 for these)

Figure 3 shows the results of our selection bias simulations, with an IV strength of r^2^ = 0.05, including all covariates in the tests of association (as results for test-r^2^perm were similar to test-indep these are provided in Supplementary Figure 3). Results for IV strength r^2^ = 0.1 and including half the covariates in the tests of association are shown in Supplementary Figure 4. The approach with the highest statistical power depended on the scenario, with the randomization test tending to have greater statistical power with low covariate correlations (usually for both the r^2^ = 0 and r^2^ = ${{N}}\left( {0, 0.1} \right)$).

The statistical power of the global randomization test changed relatively little across covariate correlations, compared with the test-r^2^perm, test-Bonf and test-indep approaches, which were sensitive to this (Fig. 3). For example, when there were 10 covariates affecting selection, 2 covariates not affecting selection and a total effect on selection of r^2^ = 0.05, power ranged between 0.254 (MCSE = 0.02) and 0.31 (MCSE = 0.02) for the global randomization test (correlation between covariates r^2^ = 0 and 0.8, respectively), and between 0.15 (MCSE = 0.02) and 0.66 (MCSE = 0.02) for the test-indep approach (correlation between covariates r^2^ = 0 and 0.8, respectively).

The statistical power of the global randomization test was well controlled (i.e., with ~5% type-1 error when no selection bias is present) across all scenarios of our selection bias simulation (see Supplementary Figure 5).

#### Using the randomization test to detect horizontal pleiotropy

**Fig. 4:**
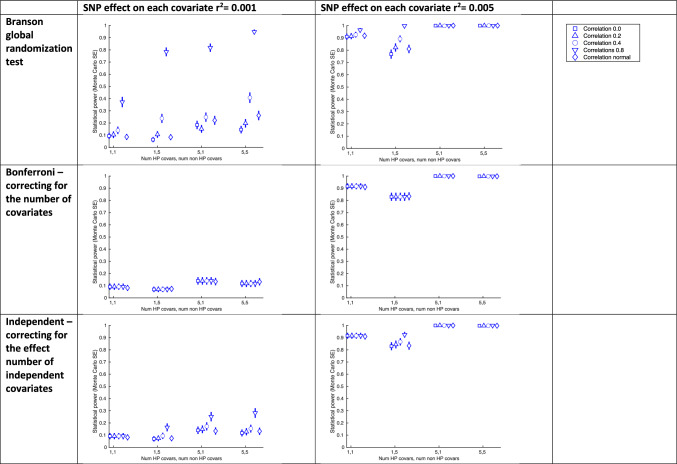
Results of horizontal pleiotropy simulations. SE: standard error. Confidence intervals are ± 1.96×MCSE (Monte Carlo standard error). Graphs show the statistical power as the number of covariates affected / not-affected by the SNP (x-axis), and the correlation between covariates (shown in legend) is varied, for each approach to identifying covariate imbalance due to selection bias. Each graph shows the results for a different test approach (rows) and strength of SNP effect on covariates (columns). In all scenarios for a SNP effect on each covariate of r^2^ = 0.01 power was at or near to 1. Results for the r^2^ permutation testing approach were very similar to the independent approach and not shown here (see Supplementary Figure 7 for these)

Figure 4 shows the results of our horizontal pleiotropy simulations. As results for test-r^2^perm were similar to test-indep these are provided in Supplementary Figure 6. In all except one simulated scenario, statistical power of the global randomization test was either comparable to the power of the alternative tests or had greater power. For example, with an effect of the horizontally pleiotropic SNP of 0.001 on each covariate, and 5 horizontal pleiotropy covariates ($$N_{chp} = 5)$$ and 5 non HP ($$N_{{c\bar{hp} }} = 5)$$, when the covariates were uncorrelated ($$r_{c}^{2} = 0)$$ we estimated similar statistical power across all approaches (e.g. 0.15 (MCSE = 0.02) and 0.12 (MCSE = 0.01) for the global randomization and test-indep approaches). In contrast, when the covariates were generated with normally distributed correlation ($$r_{c}^{2}$$ = $${{N}}\left( {0, 0.1} \right)$$), power of the global randomization was larger than the other tests (0.26 (MCSE = 0.02) compared with e.g., 0.13 (MCSE = 0.02) for the test-indep approach).

The statistical power of the global randomization test was well controlled (i.e., with ~5% type-1 error for non-horizontal pleiotropy SNPs) across all scenarios of our horizontal pleiotropy simulation (see Supplementary Figure 8).

We conducted sensitivity analyses using a less stringent P value threshold (*p* = 0.1), and as expected, statistical power is slightly higher across tests (Supplementary Figure 9).

### Applied examples

#### Detecting selection bias for CRP GRS in UK Biobank

Table 1 shows the results of our CRP selection bias analysis in UK Biobank. We did not detect an association of the CRP GRS with the restricted covariate set (containing only age and sex) using any approach (e.g., *p* = 0.813 using the global randomization test). Using the liberal covariate set we detected an association with the CRP GRS using all approaches (e.g., *p* < 0.002 and *p* = 0.010 for the global randomization test and test-r^2^-perm approaches, respectively). In contrast, we only detected an association with the CRP *cis* SNP using test-r^2^-perm (*p* = 0.004).

**Table 1:** Results of CRP selection bias applied example

		P values			
		CRP GRS		CRP *cis* SNP rs2794520	
		Restricted covariate set	Liberal covariate set	Restricted covariate set	Liberal covariate set
Global randomization test		0.813	< 0.002	0.937	0.072
r^2^ permutation test (test-r^2^perm)		0.784	0.010	0.925	0.004
Bonferroni corrected (test-Bonf)*		1.000 (2 tests)	8.88 × 10^–9^ (7 tests)	1.000 (2 tests)	0.110 (7 tests)
Independent (test-indep)*		1.000 (2 tests)	8.88 × 10^–9^ (7 tests)	1.000 (2 tests)	0.110 (7 tests)
Covariates	Age	0.840	0.840	0.923	0.923
	Sex	0.537	0.537	0.732	0.732
	Height		<0.001		0.912
	Home location—northing		<0.001		0.285
	Home location—easting		0.944		0.016
	Age completed full time education		0.587		0.222
	Townsend deprivation index		0.966		0.553

^*^P values calculated as min(1, pvalue_min_ x numTests), where pvalue_min_ is the lowest p-value of the covariates and numTests is the number of covariates for Bonferroni, and calculated with spectral decomposition for the independent version. The P values of individual traits are calculated with linear regression (IV as independent variable, covariate as dependent variable). The number of tests shown in brackets indicates the effective number of independent phenotypes in the covariate sets, i.e., the effective number of tests to use in the correction for multiple tests

#### Detecting horizontally pleiotropic CRP SNPs in UK Biobank

Of the 58 CRP-associated genetic variants, 51 were found to be associated with our defined covariate set using the global randomization test (using a threshold of *p* < 0.05). This compares to 51 identified using the test-Bonf and test-indep approaches, and 46 identified using the r^2^perm approach (see Supplementary Table 4). Using a GRS composed of all 58 SNPs, higher genetically predicted CRP levels are associated with a lower risk of CAD (odds ratio [OR]: 0.956 [95% CI: 0.918, 0.996] per 1-unit higher log CRP levels). Using a GRS composed of only the 7 SNPs not identified as horizontally pleiotropic using the global randomization test, estimates attenuated slightly to the null (OR: 0.970 [95% CI: 0.900, 1.046] per 1-unit higher log CRP levels). Results of sensitivity analyses using the *p* < 0.001 threshold were similar to the main results (see Supplementary Figure 10).

## Discussion

In this study, we have adapted a recently proposed test of association for use in MR studies. The global randomization approach tests the association of a set of covariates with a trait of interest jointly, accounting for the correlation between these covariates, rather than testing the association of the trait of interest with each covariate individually. The original study [15] proposed the global randomization test as a test for the independence assumption and assumed that the IV relevance assumption and exclusion restriction are valid. However, in an MR setting the exclusion restriction assumption (in addition to the independence assumption) may not be valid. We therefore focused on demonstrating ways in which the global randomization test can be used to identify violations of the exclusion restriction assumption, using violations due to selection bias and horizontal pleiotropy as examples. We compared the statistical power of this test to that of individual tests of the IV with each covariate with correction for the multiple tests performed using (a) Bonferroni, and (b) effective number of independent tests (calculated using spectral decomposition). In contrast to these traditional tests of covariate imbalance, the global randomization test uses a permutation-based approach to test the association of a set of covariates jointly with an IV. We also explored an alternative permutation-based approach, using the highest correlation between the covariates and IV as the test statistic.

We used simulations to investigate the statistical power of these approaches to detect selection bias and horizontal pleiotropy under different scenarios. Our selection bias simulations suggested that the global randomization test tends to have better power compared to the alternative approaches, when covariate correlations are lower. While we do not have enough information to suggest a cutoff in general, our simulations suggest the global randomization test (with MD test statistic) could be used when covariate correlations are below 0.1. In our horizontal pleiotropy simulations, the global randomization test had either similar or better power compared to the alternative approaches across all except one of the simulated scenarios. We would therefore recommend use of the global randomization test to test for horizontal pleiotropy, but we note we have not assessed every different scenario in our simulations.

We demonstrated how the global randomization test can be used in practice with two applied examples. The first sought to investigate whether MR analyses of CRP may be biased due to non-random selection in UK Biobank. We used a restricted and liberal covariate set, the former containing just age and sex, while the latter contained 5 additional variables that are unlikely to be downstream determinants of CRP genetic variants. We found evidence using all test approaches (including the global randomization test) suggesting that non-random selection may bias MR estimates of CRP. The second applied example sought to identify horizontally pleiotropic CRP-associated SNPs, when estimating the effect of CRP on coronary artery disease. The global randomization test identified 51 of the SNPs as potentially horizontally pleiotropic and estimates attenuated to the null after excluding these SNPs, although confidence intervals were wide.

Our results suggest that the global randomization test may be a useful falsification test in MR. However, one potential challenge in applying this test in this setting is the choice of covariate set. In an MR setting, most phenotypes can theoretically be downstream of a genetic variant (age and sex being two key exceptions), such that it may be difficult to identify candidate covariates to include in the global randomization test, where we believe with confidence that these covariates are not on the causal pathway between the IV and exposure, or between the exposure and outcome. In short, to use the test the researcher needs to assume that, if the IV associates with the candidate covariate set, this is more likely to be due to an invalid IV assumption rather than because these covariates are on the vertically pleiotropic pathway. Box 1 summarises the approach researchers can take to use the global randomization test to explore violations of the exclusion restriction assumption due to selection bias or horizontal pleiotropy. Where covariates may associate with the IV due to both selection bias and horizontal pleiotropy, it may be useful to first conduct a combined test for these using a combined covariate set. The value of this compared to testing for selection bias and horizontal pleiotropy separately could be investigated in future work.

**Box 1:** Overview of process to use the global randomization test

	Testing for evidence of selection bias	Testing for evidence of horizontal pleiotropy
1. Choose covariates for inclusion in covariate set	Covariates should be selected that are thought to be unaffected by the genetic instrument (and hence also not affected by the exposure phenotype), but that are potential determinants (either directly or indirectly) of selection. Included covariates cannot be perfectly correlated with selection (i.e. deterministically affect selection) as this would result in perfect balance.	Covariates should be selected that are not on the causal path between genetic instrument and exposure phenotype and are also not downstream effects of the exposure.
2. Choose test of association between genetic instrument and covariate set	If mean r^2^ between covariates <0.1: - Use global randomization test. Otherwise: - Use individual tests of each covariate with correction for the effective number of tests performed (test-indep).	Use global randomization test.
3. Choose follow-up analysis	Option 1: If confident that covariates are not consequences of the genetic instrument, conduct MR analysis conditioning on them, as this will block the pathways through which selection bias occurs hence remove the selection bias. However, this may be infeasible in cohort such as UK Biobank with complex selection mechanisms. Option 2: Conduct simulations to explore the magnitude of the selection bias that would be needed to give the observed (or null) effect, assuming no (or positive or negative effect) true effect. Option 3: Use inverse probability weighting to address selection bias, by inversely weighting the sample by the probability of selection into the sample [40], where data are available to correctly model the weights.	Include SNPs not identified as associated with the covariate set in genetic IV, and re-estimate causal effect. Compare estimate and confidence interval with estimate using all SNPs.
4. Triangulation	Test association of covariate set in a second cohort with weaker or different selection mechanism. For example, if this second cohort has less selection and the association between the IV and covariate set weakens then this strengthens the evidence for selection bias in the original cohort.	Test association of covariate set in a second cohort with weaker or different selection mechanism. For example, if this second cohort has less selection and the association between the IV and covariate set weakens then this suggests that this association is at least in part driven by selection bias rather than (solely by) horizontal pleiotropy.

In addition to the approaches using a covariate set that we focus on in this study, there are other falsification tests for exclusion restriction assumption [33, 34]. For example, heterogeneity of IV effect estimates across SNPs can be tested for, as if exclusion restriction assumption holds (in addition to the relevance and independence assumptions, and the homogeneity fourth IV assumption) the estimated effect should be consistent across IVs. Tests for this include the Hansen test, or by splitting the SNPs into distinct sets and comparing the estimated effects of the exposure using these sets [35]. While these tests are useful for looking for evidence of horizontal pleiotropy without needing to specify the set of covariates for the particular SNP, through which this pleiotropy may act, they may have low statistical power because they are comparing estimates of effect on the outcome across instruments. Steiger filtering is an approach that removes SNPs from a GRS where they explain more variation in the outcome than the exposure, such that their effect on the exposure may be more likely to be via the outcome rather than vice-versa [36]. As we have shown, the global randomization test can be used in a complementary way, to identify SNPs that may be invalid because they correlate with factors that we do not believe could be on the causal pathway (i.e., between IV and exposure or between exposure and outcome), and hence can be removed from a GRS. This ‘randomization filtering’ can be used as a sensitivity analysis in MR studies.

Our study has a number of strengths and limitations. Strengths include the fact that we explored the value of the global randomization test using both simulations and applied examples. We tried to simulate realistic scenarios by basing aspects such as the recruitment rate on real data (in this case UK Biobank). However, we were only able to simulate a limited number of scenarios, such that we cannot infer how the statistical power of the global randomization test compares to the alternative approaches beyond these. We generalized the Mahalanobis distance used in [15] to allow continuous and ordinal instruments as well as binary. Our approach assumes a linear relationship between the IV (including GRS and SNP dosages) and each of the covariates, such that non-linear associations may have been missed. As with any approach assuming linearity, researchers can examine this assumption (e.g. by plotting the observed data), then, if this suggests a non-linear relationship may exist, they could adapt the approach to test for this. We used the global randomization test to test the association of a single genetic variant or independent SNPs combined into a GRS, with a covariate set. It may be possible to extend this to incorporate multiple correlated SNPs, for example, those used in *cis*-MR studies [37]. We did not adjust for any covariates (e.g., genetic principal components) in our examples, but in future work using this approach the genetic instrument and covariates could be regressed on potential confounders and then the residuals from those regressions can be used in the test process. In simulations we used a P value threshold of $0.05$ to calculate the rejection percentage, but in practice researchers should avoid using P value thresholds to determine ‘statistical significance’ (or ‘hits’) where possible. Where it is necessary to use a threshold, researchers may choose to use a different threshold to 0.05. For example, when identifying SNPs to exclude in a sensitivity analysis because they may be horizontally pleiotropic, a less stringent threshold may be preferred to exclude SNPs where there is even a small amount of evidence that they are horizontally pleiotropic. While our scenarios use a single time-fixed exposure with no exposure-confounder feedback, where exposures are time-varying or have exposure-confounder feedback the implications of our study are the same assuming effect estimates are interpreted as an effect of underlying exposure liability (see DAGs in Supplementary Figures 12 and 13) [38].

In summary, the global randomization test can be used as a first step for identifying potential violations of Mendelian randomization assumptions. Where an association is identified that suggests horizontal pleiotropy, a researcher can then investigate this further, for example, by using instruments for variables in the covariate set to test for an effect of these on the outcome of interest [39]. The choice of covariate set used with the global randomization test needs careful consideration in the context of the specific exposure and outcome being examined. While we have focused on falsification tests for the exclusion restriction assumption, this approach may also be useful as a falsification test for the independence assumption, for example, testing for confounding via dynastic effects. In practice it may be difficult to determine which of these assumptions is violated when an association is identified between an IV and covariate set.

## Supplementary Information

Below is the link to the electronic supplementary material.

Supplementary file1 (PDF 1895 KB)

## References

[1] Davey Smith G, Ebrahim S. “Mendelian randomization”: can genetic epidemiology contribute to understanding environmental determinants of disease? Int J Epidemiol. 2003;32:1–22. 12689998 10.1093/ije/dyg070

[2] Lawlor DA, Harbord RM, Sterne JAC, Timpson N, Davey SG. Mendelian randomization: using genes as instruments for making causal inferences in epidemiology. Stat Med. 2008;27:1133–63. 17886233 10.1002/sim.3034

[3] Richmond RC, Davey SG. Mendelian randomization: concepts and scope. Cold Spring Harb Perspect Medi. 2022;12: a040501. 10.1101/cshperspect.a040501 PMC8725623 34426474

[4] Sanderson E, Glymour MM, Holmes MV, et al. Mendelian randomization Nat Rev Methods Primers. 2022;2:6. 37325194 10.1038/s43586-021-00092-5 PMC7614635

[5] Hemani G, Bowden J, Davey SG. Evaluating the potential role of pleiotropy in Mendelian randomization studies. Hum Mol Genet. 2018;27:R195-208. 29771313 10.1093/hmg/ddy163 PMC6061876

[6] Lousdal ML. An introduction to instrumental variable assumptions, validation and estimation. Emerg Themes Epidemiol. 2018;15:1. 29387137 10.1186/s12982-018-0069-7 PMC5776781

[7] Brumpton B, Sanderson E, Heilbron K, et al. Avoiding dynastic, assortative mating, and population stratification biases in Mendelian randomization through within-family analyses. Nat Commun. 2020;11:3519. 32665587 10.1038/s41467-020-17117-4 PMC7360778

[8] Pirastu N, Cordioli M, Nandakumar P, et al. Genetic analyses identify widespread sex-differential participation bias. Nat Genet. 2021;53:663–71. 33888908 10.1038/s41588-021-00846-7 PMC7611642

[9] Jackson JW, Swanson SA. Toward a clearer portrayal of confounding bias in instrumental variable applications. Epidemiol. 2015;26:498–504. 10.1097/EDE.0000000000000287 PMC4673662 25978796

[10] C Reactive Protein Coronary Heart Disease Genetics Collaboration. Association between C reactive protein and coronary heart disease: Mendelian randomisation analysis based on individual participant data. BMJ. 2011;342:d548. 10.1136/bmj.d548 PMC3039696 21325005

[11] Davey Smith G, Lawlor DA, Harbord R, et al. Association of C-reactive protein with blood pressure and hypertension. Arterioscler Thromb Vasc Biol. 2005;25:1051–6. 15731495 10.1161/01.ATV.0000160351.95181.d0

[12] Davies NM, Thomas KH, Taylor AE, et al. How to compare instrumental variable and conventional regression analyses using negative controls and bias plots. Int J Epidemiol. 2017;46:2067–77. 28398582 10.1093/ije/dyx014 PMC5837536

[13] Mountjoy E, Davies NM, Plotnikov D, et al. Education and myopia: assessing the direction of causality by mendelian randomisation. BMJ. 2018;361: k2022. 29875094 10.1136/bmj.k2022 PMC5987847

[14] Davies NM, Dickson M, Davey Smith G, et al. The effect of education on adult mortality, health, and income: triangulating across genetic and policy reforms. bioRxiv. 2018;250068.

[15] Branson Z, Keele L. Evaluating a key instrumental variable assumption using randomization tests. Am J Epidemiol. 2020;189:1412–20. 32432319 10.1093/aje/kwaa089

[16] Franklin JM, Rassen JA, Ackermann D, et al. Metrics for covariate balance in cohort studies of causal effects. Stat Med. 2014;33:1685–99. 24323618 10.1002/sim.6058

[17] Uddin MJ, Groenwold RHH, de Boer A, et al. Evaluating different physician’s prescribing preference based instrumental variables in two primary care databases: a study of inhaled long-acting beta2-agonist use and the risk of myocardial infarction. Pharmacoepidemiol Drug Saf. 2016;25:132–41. 27038359 10.1002/pds.3860

[18] Fang G, Brooks JM, Chrischilles EA. Comparison of instrumental variable analysis using a new instrument with risk adjustment methods to reduce confounding by indication. Am J Epidemiol. 2012;175:1142–51. 22510277 10.1093/aje/kwr448

[19] Rassen JA, Brookhart MA, Glynn RJ, et al. Instrumental variables II: instrumental variable application—in 25 variations, the physician prescribing preference generally was strong and reduced covariate imbalance. J Clin Epidemiol. 2009;62:1233–41. 19345561 10.1016/j.jclinepi.2008.12.006 PMC2886011

[20] Huybrechts KF, Gerhard T, Franklin JM, et al. Instrumental variable applications using nursing home prescribing preferences in comparative effectiveness research. Pharmacoepidemiol Drug Saf. 2014;23:830–8. 24664805 10.1002/pds.3611 PMC4116440

[21] Davies NM, Gunnell D, Thomas KH, et al. Physicians’ prescribing preferences were a potential instrument for patients’ actual prescriptions of antidepressants. J Clin Epidemiol. 2013;66:1386–96. 24075596 10.1016/j.jclinepi.2013.06.008 PMC3824069

[22] Fang G, Brooks JM, Chrischilles EA. A new method to isolate local-area practice styles in prescription use as the basis for instrumental variables in comparative effectiveness research. Med Care. 2010;48:710–7. 20613655 10.1097/MLR.0b013e3181e41bb2

[23] Morris TP, White IR, Crowther MJ. Using simulation studies to evaluate statistical methods. Stat Med. 2019;38:2074–102. 30652356 10.1002/sim.8086 PMC6492164

[24] Swanson JM. The UK biobank and selection bias. Lancet. 2012;380:110. 22794246 10.1016/S0140-6736(12)61179-9

[25] Davey Smith G, Lawlor DA, Harbord R, et al. Clustered environments and randomized genes: a fundamental distinction between conventional and genetic epidemiology. PLOS Med. 2007;4: e352. 18076282 10.1371/journal.pmed.0040352 PMC2121108

[26] Zheng J, Richardson TG, Millard LAC, et al. PhenoSpD: an integrated toolkit for phenotypic correlation estimation and multiple testing correction using GWAS summary statistics. GigaSci. 2018;7:giy090. 10.1093/gigascience/giy090 PMC6109640 30165448

[27] Li J, Ji L. Adjusting multiple testing in multilocus analyses using the eigenvalues of a correlation matrix. Heredity. 2005;95:221–7. 16077740 10.1038/sj.hdy.6800717

[28] Nyholt DR. A simple correction for multiple testing for single-nucleotide polymorphisms in linkage disequilibrium with each other. Am J Hum Genet. 2004;74:765–9. 14997420 10.1086/383251 PMC1181954

[29] Mitchell R, Hemani G, Dudding T, et al. UK Biobank genetic data: MRC-IEU quality control, version 2. 2019.

[30] Ligthart S, Vaez A, Võsa U, et al. Genome analyses of >200,000 individuals identify 58 loci for chronic inflammation and highlight pathways that link inflammation and complex disorders. Am J Hum Genet. 2018;103:691–706. 30388399 10.1016/j.ajhg.2018.09.009 PMC6218410

[31] Taylor AE, Jones HJ, Sallis H, et al. Exploring the association of genetic factors with participation in the Avon longitudinal study of parents and children. Int J Epidemiol. 2018;47:1207–16. 29800128 10.1093/ije/dyy060 PMC6124613

[32] Rassen JA, Schneeweiss S, Glynn RJ, et al. Instrumental variable analysis for estimation of treatment effects with dichotomous outcomes. Am J Epidemiol. 2008;169:273–84. 19033525 10.1093/aje/kwn299

[33] Labrecque J, Swanson SA. Understanding the assumptions underlying instrumental variable analyses: a brief review of falsification strategies and related tools. Curr Epidemiol Rep. 2018;5:214–20. 30148040 10.1007/s40471-018-0152-1 PMC6096851

[34] Skrivankova VW, Richmond RC, Woolf BAR, et al. Strengthening the reporting of observational studies in epidemiology using mendelian randomisation (STROBE-MR): explanation and elaboration. BMJ. 2021;375: n2233. 34702754 10.1136/bmj.n2233 PMC8546498

[35] Millard LAC, Davies NM, Tilling K, et al. Searching for the causal effects of BMI in over 300 000 individuals, using Mendelian randomization. PLOS Genet. 2019;15: e1007951. 30707692 10.1371/journal.pgen.1007951 PMC6373977

[36] Hemani G, Tilling K, Davey SG. Orienting the causal relationship between imprecisely measured traits using GWAS summary data. PLOS Genet. 2017;13: e1007081. 29149188 10.1371/journal.pgen.1007081 PMC5711033

[37] Gkatzionis A, Burgess S, Newcombe PJ. Statistical methods for cis-Mendelian randomization with two-sample summary-level data. Genet Epidemiol. 2023;47:3–25. 36273411 10.1002/gepi.22506 PMC7614127

[38] Morris TT, Heron J, Sanderson ECM, et al. Interpretation of Mendelian randomization using a single measure of an exposure that varies over time. Int J Epidemiol. 2022;51:1899–909. 35848950 10.1093/ije/dyac136 PMC9749705

[39] Yang Q, Sanderson E, Tilling K, et al. Exploring and mitigating potential bias when genetic instrumental variables are associated with multiple non-exposure traits in Mendelian randomization. Eur J Epidemiol. 2022;37:683–700. 35622304 10.1007/s10654-022-00874-5 PMC9329407

[40] Gkatzionis A, Burgess S. Contextualizing selection bias in Mendelian randomization: how bad is it likely to be? Int J Epidemiol. 2019;48:691–701. 30325422 10.1093/ije/dyy202 PMC6659463

[41] Swanson SA. A practical guide to selection bias in instrumental variable analyses. Epidemiol. 2019;30:345–49. 10.1097/EDE.0000000000000973 30896458

