# Supplementary material for: Using the global randomization test as a Mendelian randomization falsification test for the exclusion restriction assumption
Source: Eur J Epidemiol. 2024 Feb 29;39(8):843–55. doi: 10.1007/s10654-024-01097-6 (PMC11410989; doi:10.1007/s10654-024-01097-6)
Supplement: Supplementary file 1 — Supplementary file1 (PDF 1895 KB) [file 10654_2024_1097_MOESM1_ESM.pdf]

Using the global randomization test as A Mendelian randomization falsification test for the exclusion restriction assumption

Millard et al.

SUPPLEMENTARY INFORMATION

TABLE OF CONTENTS

SUPPLEMENTARY TEXT 3

Supplementary section S1: Comparing speed of correlation and regression approaches to calculating mean difference. 3

Supplementary section S2: Details of selection bias simulation data generating mechanism 3

Supplementary section S3: Simulation performance measure 5

Supplementary section S4: Details of covariates used in horizontal pleiotropy applied example 5

Supplementary section S5: Derivation of coronary heart disease phenotype 5

SUPPLEMENTARY FIGURES 6

Supplementary figure 1: DAG for simulations with parameters used 6

Supplementary figure 2: Sample for applied UK Biobank examples 7

Supplementary figure 3: Results of selection bias simulations for instrument strength r^2=0.05 and all covariates included in test, including r^2 permutation testing approach 8

Supplementary figure 4: Results of selection bias simulations 9

Supplementary figure 5: Results of selection bias simulations for instrument strength r^2=0.05 and all covariates included in test, using the whole sample 12

Supplementary figure 6: Covariate x Exposure interaction sizes for selection bias simulations with instrument strength r^2=0.05 13

Supplementary figure 7: Results of horizontal pleiotropy simulations, including r^2 permutation testing approach 14

Supplementary figure 8: Results of horizontal pleiotropy simulations for non-horizontally pleiotropic SNP (with effect of the horizontally pleiotropic SNP on the covariates set to r^2=0.001 15

Supplementary figure 9: Results of simulation sensitivity analyses using P value threshold of 0.1 16

Supplementary figure 10: Results of horizontal pleiotropy applied example 17

Supplementary figure 11: Relationship between number of tests and Bonferroni / independent test P value thresholds 18
Supplementary figure 12: DAGs showing equivalent scenarios for time-varying exposures 19
Supplementary figure 13: DAGs showing scenarios for time-varying exposures with exposure-confounder feedback 20
SUPPLEMENTARY TABLES 21
Supplementary table 1: Correlations between covariates used to assess selection bias in MR applied analysis 21
Supplementary table 2: Correlations between covariates used in horizontal pleiotropy applied example 22
Supplementary table 3: P values for individual tests of covariates with SNPs for horizontal pleiotropy applied example 23
Supplementary table 4: Results of horizontal pleiotropy applied example – identifying horizontally pleiotropic CRP SNPs 25
REFERENCES 27

SUPPLEMENTARY TEXT

Supplementary section S1: Comparing speed of correlation and regression approaches to calculating mean difference.

We found the correlation approach to calculating mean difference was ~12 times faster than using linear regression (lm R function). We obtained this by running the approaches 10 times assuming 920,000 participants and normally distributed variables, and calculating the average time each approach took across these. This test was conducted on a single core of a Lenovo nx360 m5 compute node with a 2.4 GHz Intel E5-2680 v4 CPU, on the University of Bristol's BlueCrystal phase 4 high-performance computing service. The code for this test can be found at: https://github.com/MRCIEU/MR-randomization-test/tree/main/1-sims/generic-functions/speedTest

Supplementary section S2: Details of selection bias simulation data generating mechanism

Controlling the variance of X explained by C_s and Z

To control the variance of X explained by C_s and Z, we first generate an intermediate variable that combines the covariates in C_s:

$$I_{CS} = \sum_{i=1}^{N_{cs}} C_s\,(i)$$

Then, X is generated as:

$$X = \beta_z Z + \beta_{ICS} I_{CS} + \ \beta_{\in} \in$$

Where ∈ is normally distributed random error.

The total variance of X can be defined in terms of the variances and covariances of Z and I_{CS}:

$$var(total) = {\beta_z}^2 var(Z) + {\beta_{ICS}}^2 var(I_{CS}) + \ 2\beta_z \beta_{ICS} \mathrm{cov}(\mathrm{Z}, I_{CS})$$

And given that Z and C are independent $\mathrm{cov}(\mathrm{Z}, I_{CS}) = 0$:

$$var(total) = {\beta_z}^2 var(Z) + {\beta_{ICS}}^2 var(I_{CS})$$

We fix the variance of X explained by Z (i.e. the total effect of Z on X), to $r_{zx}^2 \in \{0.05, 0.1\}$:

$$\beta_z = \sqrt{r_{zx}^2}$$

Then set $\beta = \beta_{ICS}$ such that the total variance explained by I_{CS} is 10%:

$$0.1 = \beta^2 var(I_{CS})$$

$$\beta = \sqrt{\frac{0.1}{var(I_{CS}))}}$$

We set the variance of X to 1, so set the variance of X explained by the error term to:

$$\beta_{\in} = \sqrt{(1-(r_{zx}^2+0.1))}.$$

Controlling the variance of S explained by C_s and X

For the selection binary variable S, we also fix the total effects. We assumed a total effect of X and C_s on S of r^2 = {0.05, 0.1, 0.2}. We assume 5.5% are selected into our sample (the proportion of those invited who agreed to participate [1]). To generate S we first generate intermediate covariate I_{CS}:

$$I_{CS} = \sum_{i=1}^{N_{cs}} C_s\,(i)$$

This defines that each covariate has the same effect on S (I.e. a linear function of the covariates on intermediate variable I_{CS} with $\beta_i = 1$).

We then generate a continuous selection variable, S_{cont}, with mean zero and sd=1.

The total variance of S_{cont} can be defined in terms of the variances and covariances of X and I_{CS}:

$$var(total) = {\beta_X}^2 var(X) + {\beta_{ICS}}^2 var(I_{CS}) + 2\beta_X \beta_{ICS} \mathrm{cov}(\mathrm{X}, I_{CS})$$

we are assuming X and I_{CS} explain 100% of the variance of S_{cont}, and an equal effect of X versus C_s on S_{cont}, such that:

$$var(total) = 1 = \beta^2 var(X) + \beta^2 var(I_{CS}) + 2\beta^2 \mathrm{cov}(\mathrm{X}, I_{CS})$$

$$\beta = \sqrt{\frac{1}{var(X) + var(I_{CS}) + 2(\mathrm{cov}(\mathrm{X}, I_{CS}))}}$$

We then generate S_{cont}:

$$S_{cont} = \beta x + \beta I_{CS}$$

We then generate S using S_{cont}, changing the odds ratio and intercept in this model accordingly to give r^2 = {0.05, 0.1, 0.2}, where the probability of being selected, P_S, is given by:

$$P_S = \frac{\exp(log(OR) \times S_{cont} + intercept)}{1 + \exp\,(\log(OR) \times S_{cont} + intercept)}$$

Supplementary section S3: Simulation performance measure

We will evaluate statistical power using rejection percentage [2]:

$$RP = \frac{1}{n_{sim}} \sum_{i=1}^{n_{sim}} 1(p_i \leq 0.05)$$

Where p_i is the p-value of simulation iteration *I,* and n_{sim} is the number of simulation repetitions, which we set to 500.

Monte Carlo standard error (SE) is estimated as:

$$\sqrt{\frac{RP \times (1 - RP)}{n_{sim}}}$$

Supplementary section S4: Details of covariates used in horizontal pleiotropy applied example

Weight and height were both measured at baseline assessment. Weight was measured (to the nearest 100 g) in light clothing and unshod using a Tanita BC418MA body composition analyser and height to the nearest cm using a Seca 202 device. We used the mean SBP and DBP, respectively, from two resting automated measures, measured using an Omron HEM-7105IT digital blood pressure monitor. Blood samples were collected at baseline and total cholesterol, HDL cholesterol, apolipoprotein A1, apolipoprotein B, albumin, lipoprotein A, leukocyte count and glucose were measured by immunoturbidimetric analysis on a Beckman Coulter AU5800. Waist and hip circumference were measured at baseline and used to derive waist hip ratio. Age participants started smoking, stopped smoking and the number of cigarettes smoked per day were used to derive a measure of smoking pack years, with those who have never smoked assigned the value zero.

Supplementary section S5: Derivation of coronary heart disease phenotype

We derived a binary variable denoting coronary heart disease, where participants with a date of first occurrence for any of the following diseases were assigned as having coronary artery disease:

- Angina pectoris (UK Biobank field ID 131296)
- Acute myocardial infarction (UK Biobank field ID 131298)
- Subsequent myocardial infarction (UK Biobank field ID 131300)
- Certain current complications following acute myocardial infarction (UK Biobank field ID 131302)
- Other acute ischaemic heart diseases (UK Biobank field ID 131304)
- Chronic ischaemic heart disease (UK Biobank field ID 131306)

SUPPLEMENTARY FIGURES

Supplementary figure 1: DAG for simulations with parameters used

a) Selection bias

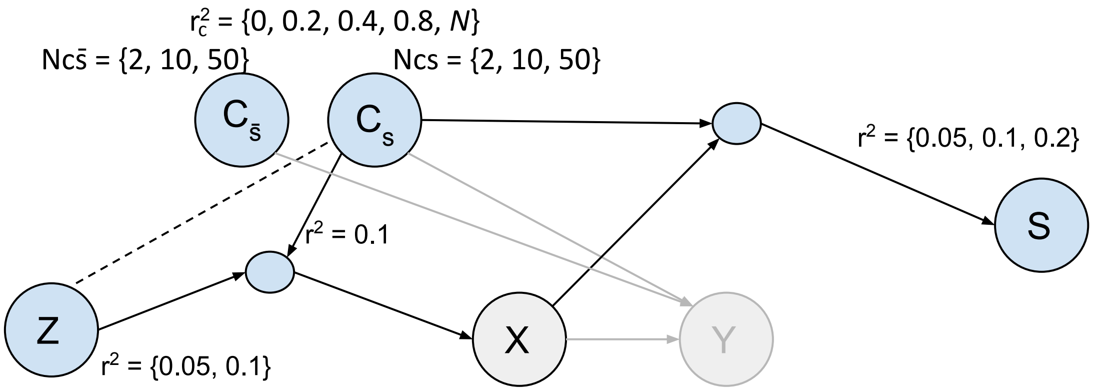

b) Horizontal pleiotropy

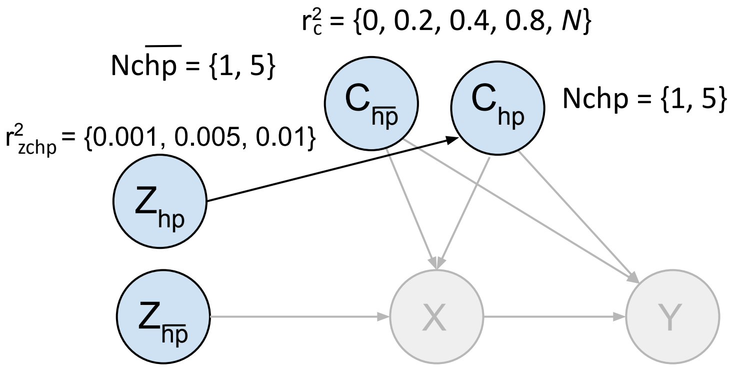

DAGs show the simulation parameters used in a fully factorial design. The grey edges and nodes are those that do not impact the simulation, so the relationships depicted by these edges do not need to be defined and the variables depicted by these nodes do not need to be simulated.

Supplementary figure 2: Sample for applied UK Biobank examples

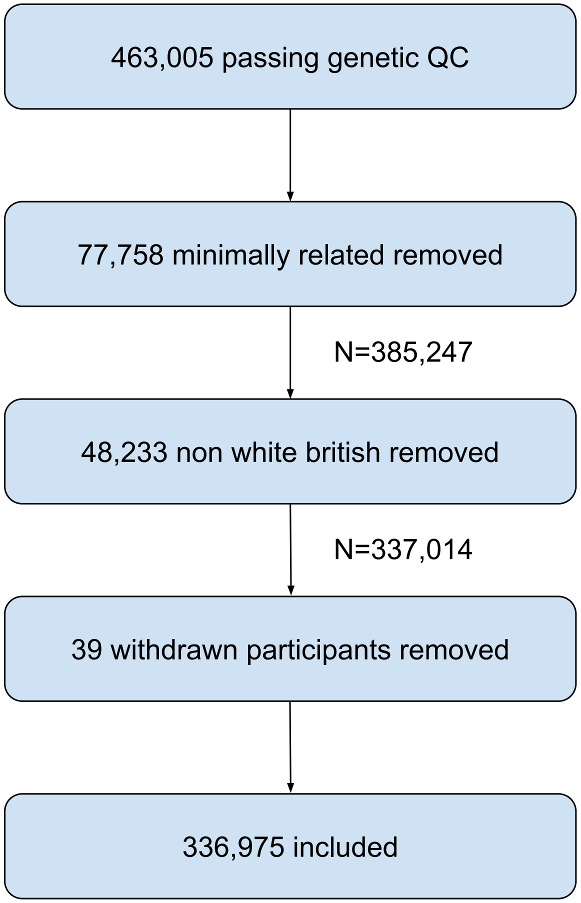

Supplementary figure 3: Results of selection bias simulations for instrument strength r^2=0.05 and all covariates included in test, including r^2 permutation testing approach

**Total effect on selection**	**Number of covariates affecting selection $(N_{cs}) = 2$**	**Number of covariates affecting selection $(N_{cs}) = 10$**	**Number of covariates affecting selection $(N_{cs}) = 50$**
**r^2=0.05**	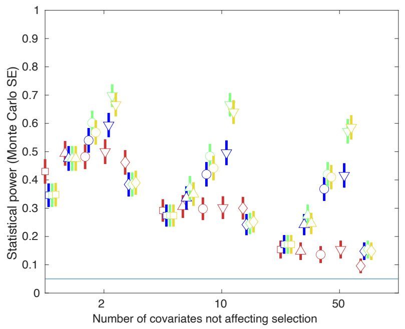	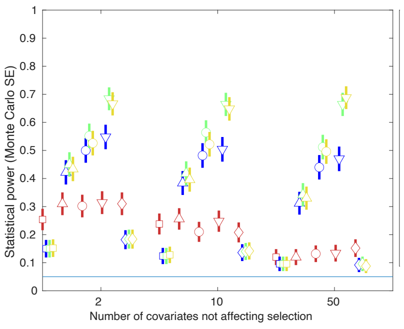	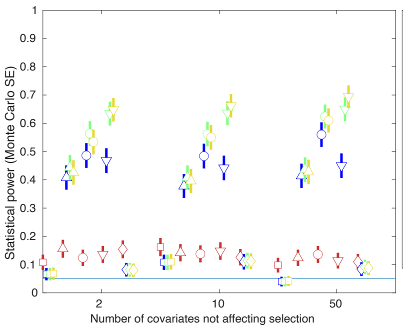
**r^2=0.1**	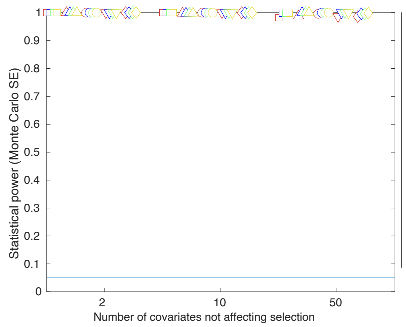	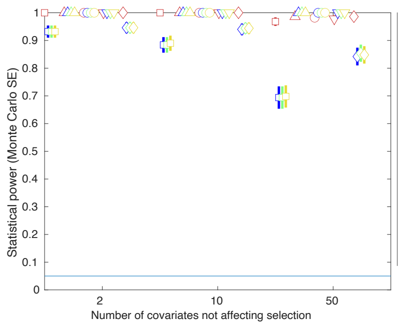	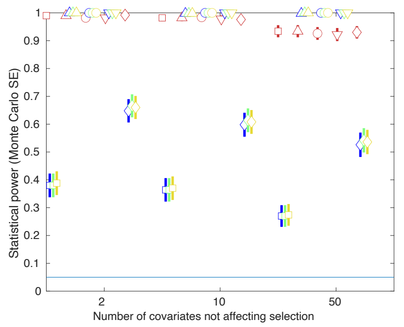

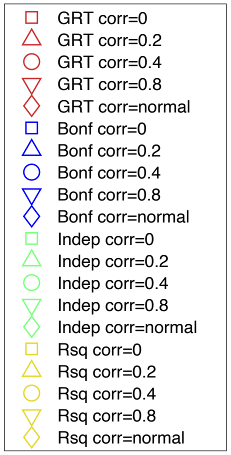

GRT: global randomization test; SE: standard error. Total effect on selection: the total effect of covariates C_S and X on selection S. Confidence intervals are +/- 1.96*MCSE (Monte Carlo standard error). Each graph shows the statistical power of each approach to identifying covariate imbalance due to selection bias, as the number of covariates not affecting selection (x-axis) and the correlation between covariates (shown in legend) are varied. Each graph shows the results for a particular number of covariates affecting selection (N_{cs}; columns), and total effect of covariates C_S and X on selection (r^2; rows). The plots compare the power of the GRT, with the Bonferroni correction ('Bonf' in legend), correction for the equivalent number of independent covariates ('Indep' in legend) and an alternative r^2 permutation testing approach ('Rsq' in legend). When r^2=0.2 for the total effect on selection, power was at or near 1 in all scenarios.

Supplementary figure 4: Results of selection bias simulations

a) Instrument strength rsq=0.05, half covariates included in test

Total effect on selection	$N_{cs} = 2$	$N_{cs} = 10$	$N_{cs} = 50$
R^2=0.05	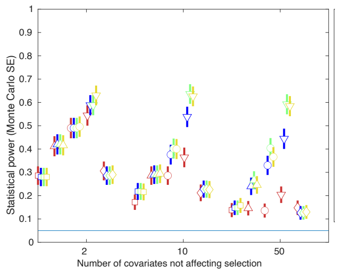	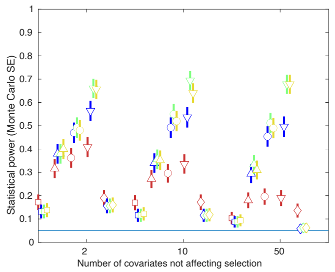	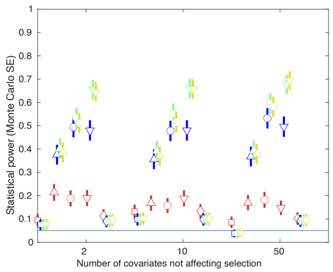
R^2=0.1	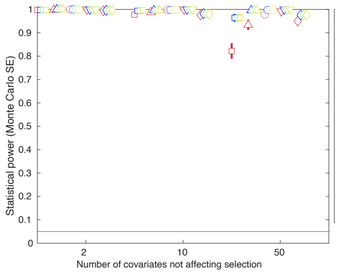	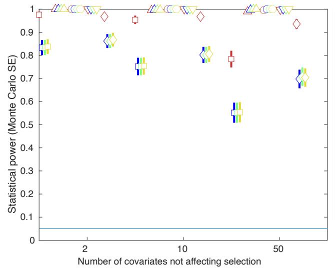	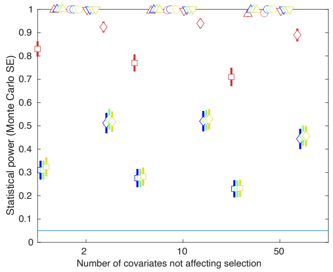
R^2=0.2	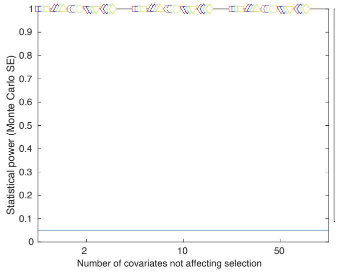	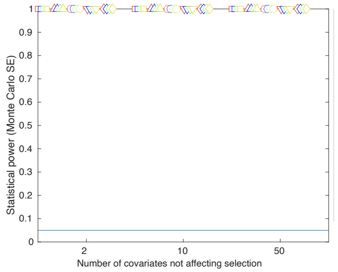	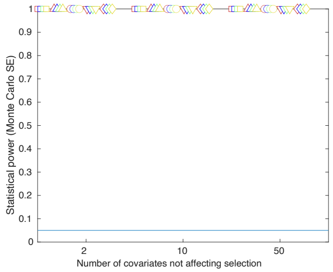

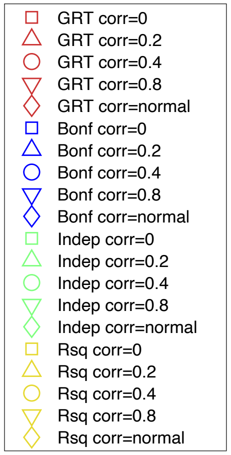

b) Instrument strength rsq=0.1, all covariates included in test

**Total effect on selection**	$N_{cs} = 2$	$N_{cs} = 10$	$N_{cs} = 50$
**R^2=0.05**	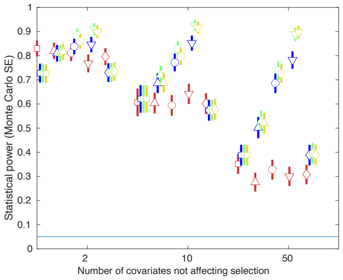	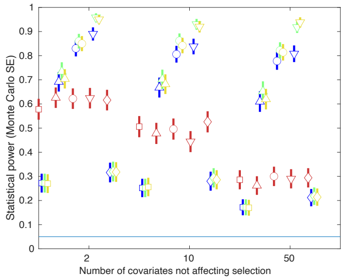	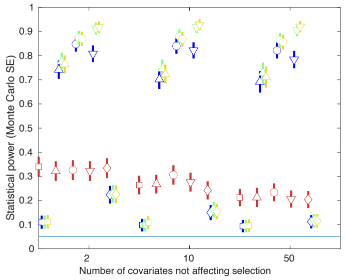
**R^2=0.1**	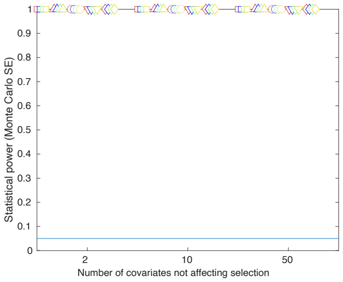	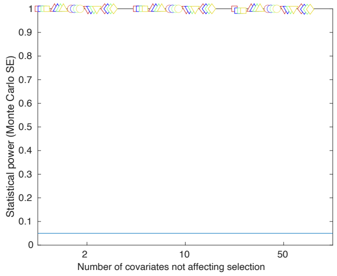	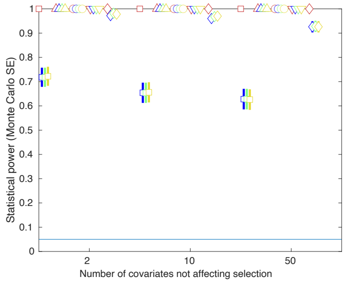
**R^2=0.2**	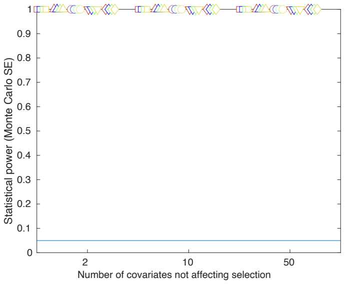	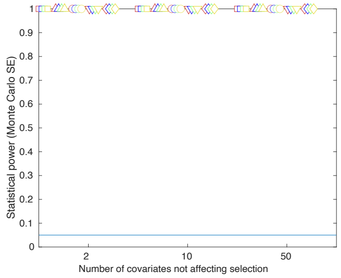	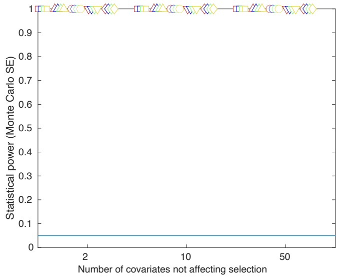

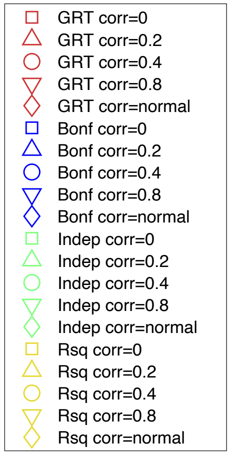

c) Instrument strength rsq=0.1, half covariates included in test

Total effect on selection	$N_{cs} = 2$	$N_{cs} = 10$	$N_{cs} = 50$
R^2=0.05	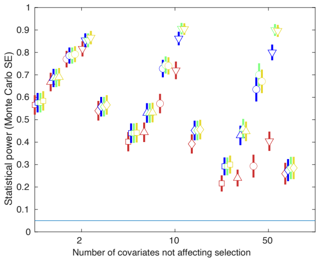	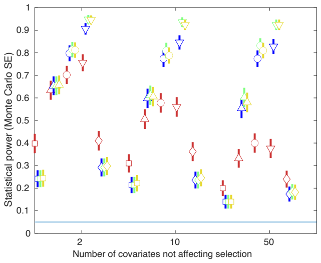	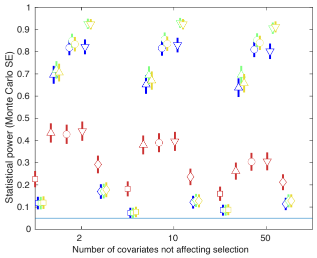
R^2=0.1	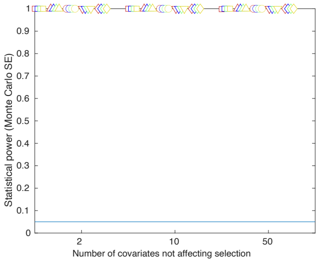	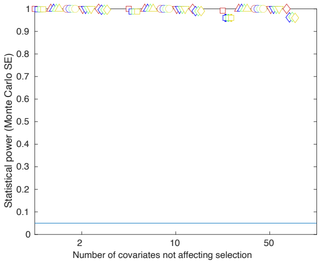	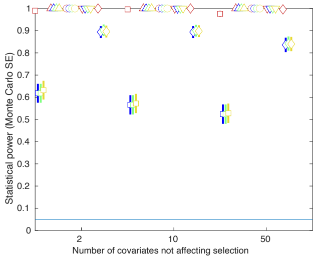
R^2=0.2	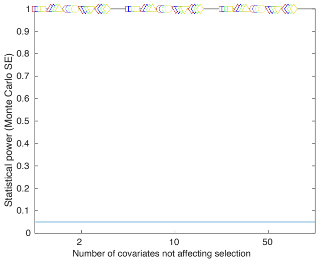	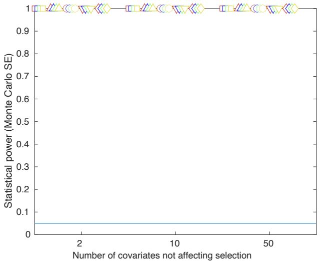	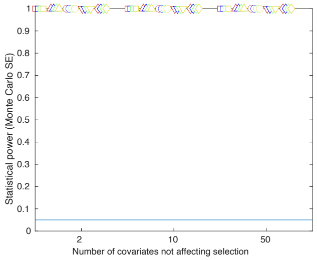

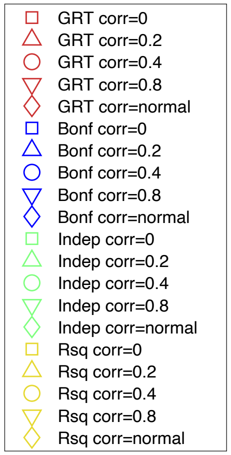

GRT: global randomization test; SE: standard error. Total effect on selection: the total effect of covariates C_S and X on selection S. N_{cs}: The number of Covariates affecting selection. Confidence intervals are +/- 1.96*MCSE (Monte Carlo standard error).

Supplementary figure 5: Results of selection bias simulations for instrument strength r^2=0.05 and all covariates included in test, using the whole sample

**Total effect on selection**	$N_{cs} = 2$	$N_{cs} = 10$	$N_{cs} = 50$
**R^2=0.05**	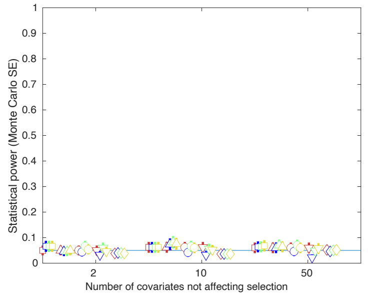	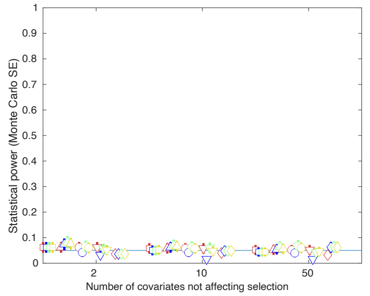	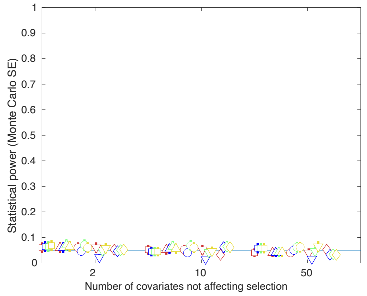
**R^2=0.1**	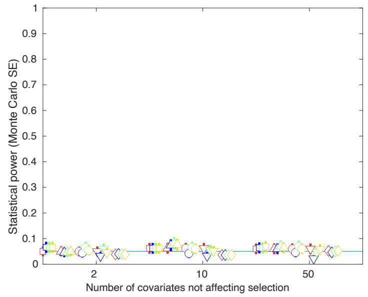	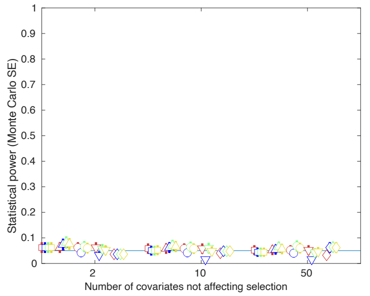	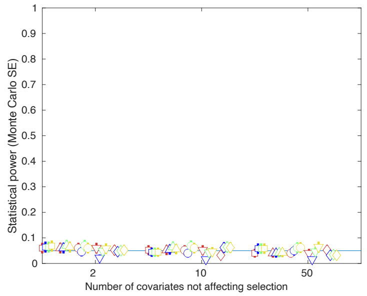
**R^2=0.2**	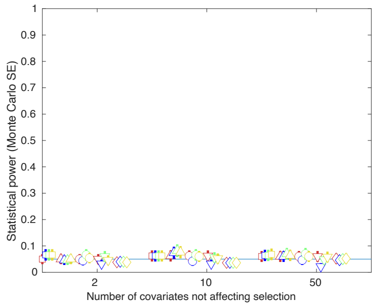	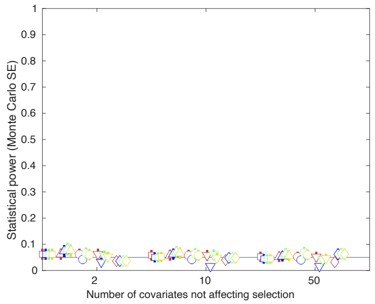	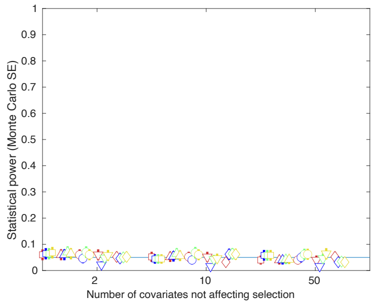

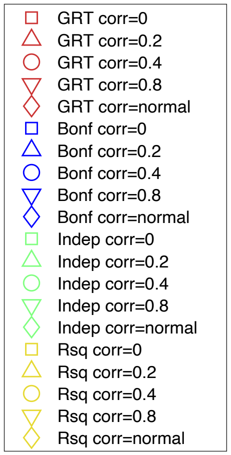

GRT: global randomization test; SE: standard error. Total effect on selection: the total effect of covariates C_S and X on selection S. N_{cs}: The number of Covariates affecting selection. Confidence intervals are +/- 1.96*MCSE (Monte Carlo standard error).

Supplementary figure 6: Covariate x Exposure interaction sizes for selection bias simulations with instrument strength r^2=0.05

<table>
<tr><th>Total effect on selection</th><th>$N_{cs} = 2$</th><th>$N_{cs} = 10$</th><th>$N_{cs} = 50$</th></tr>
<tr><td>R^2=0.05</td>
<td>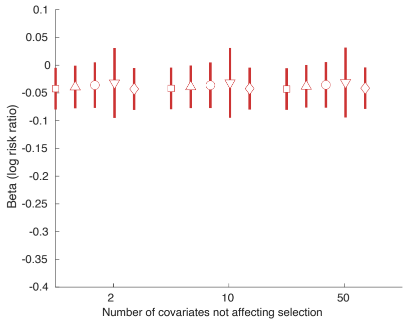
</td>
<td>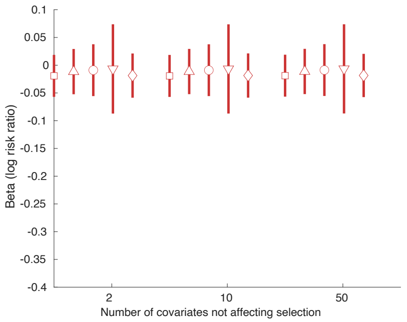
</td>
<td>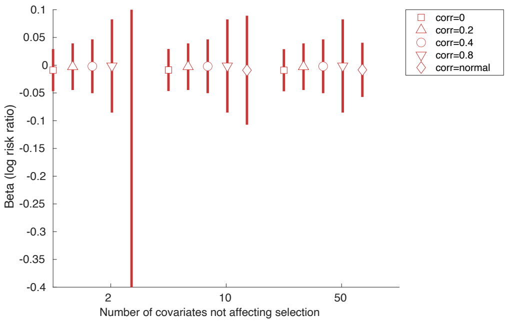
</td></tr>
<tr><td>R^2=0.1</td>
<td>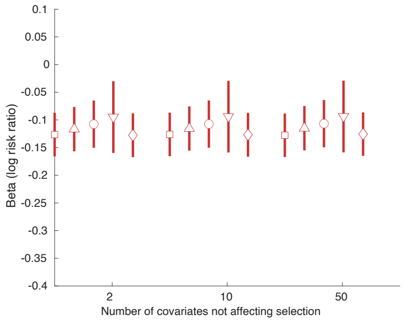
</td>
<td>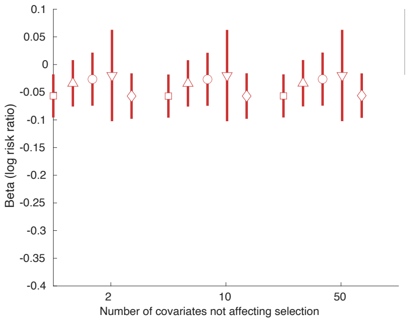
</td>
<td>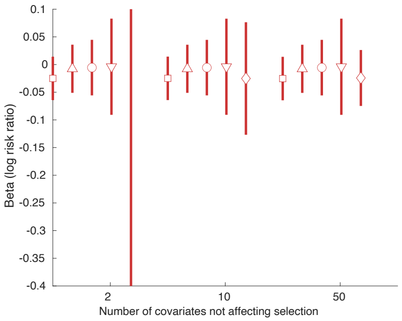
</td></tr>
<tr><td>R^2=0.2</td>
<td>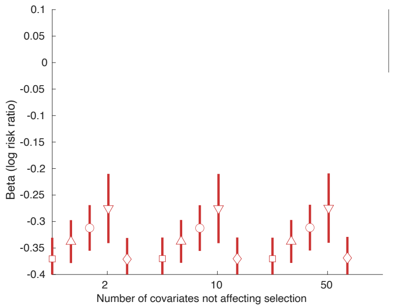
</td>
<td>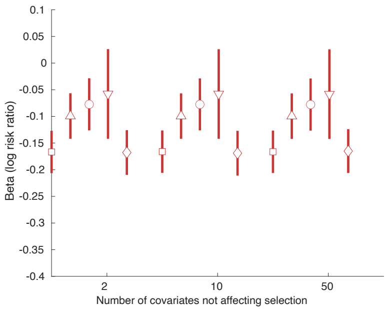
</td>
<td>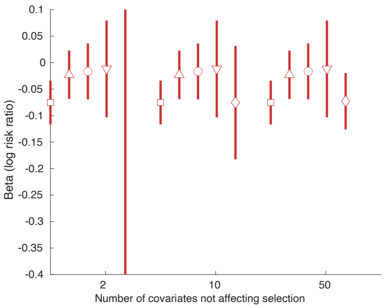
</td></tr>
</table>

Average coefficient (1.96x model-based SE [i.e. average SE across all models]) for interaction terms in a poisson regression model estimating the association of covariates C_S, and each of their interactions with exposure x, with selection. N_{cs}: The number of Covariates affecting selection. Confidence intervals are +/- 1.96*MCSE (Monte Carlo standard error).

Supplementary figure 7: Results of horizontal pleiotropy simulations, including r^2 permutation testing approach

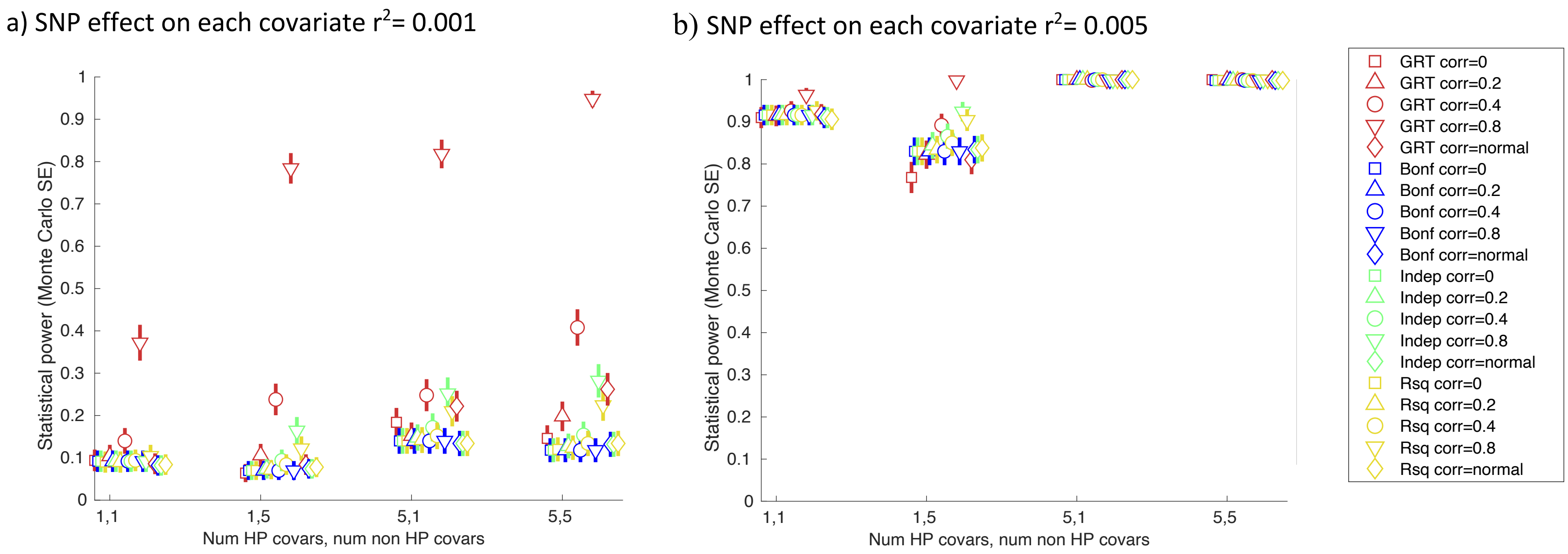

GRT: global randomization test; SE: standard error. Confidence intervals are +/- 1.96*MCSE (Monte Carlo standard error). Graphs show the statistical power as the number of covariates affected / not-affected by the SNP (x-axis), and the correlation between covariates (shown in legend) is varied, for each approach to identifying covariate imbalance due to selection bias (shown in legend). Each graph shows the results for a different strength of SNP effect on covariates. The plots compare the power of the GRT, with the Bonferroni correction ('Bonf' in legend), correction for the equivalent number of independent covariates ('Indep' in legend) and an alternative r^2 permutation testing approach ('Rsq' in legend). In all scenarios for a SNP effect on each covariate of r^2=0.01 power was at or near to 1.

Supplementary figure 8: Results of horizontal pleiotropy simulations for non-horizontally pleiotropic SNP (with effect of the horizontally pleiotropic SNP on the covariates set to r^2=0.001

a) SNP effect on each covariate R^2= 0.001

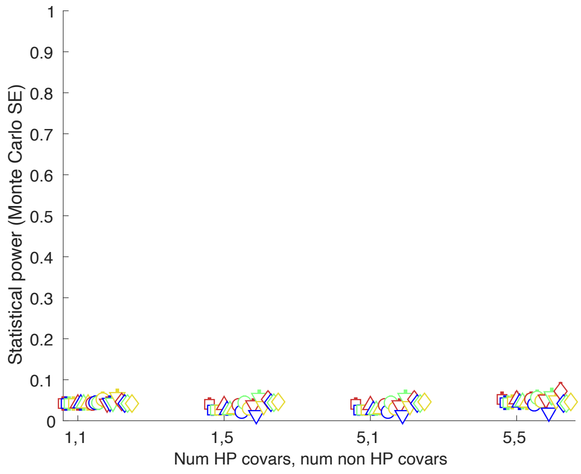

b) SNP effect on each covariate R^2= 0.005

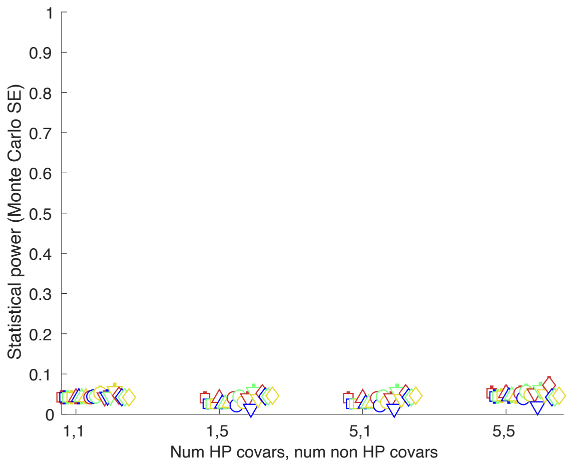

c) SNP effect on each covariate R^2= 0.01

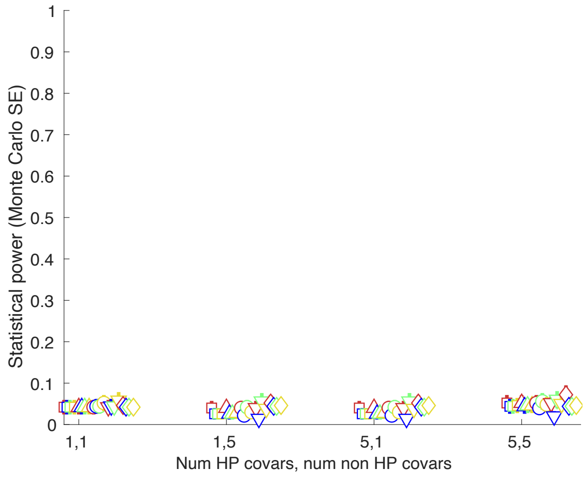

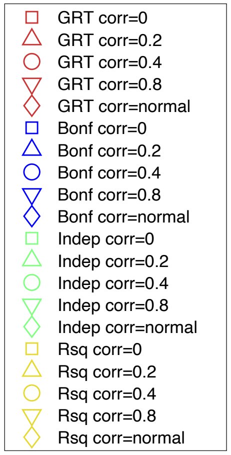

GRT: global randomization test; SE: standard error. Confidence intervals are +/- 1.96*MCSE (Monte Carlo standard error).

Supplementary figure 9: Results of simulation sensitivity analyses using P value threshold of 0.1

a) Selection bias simulation: Number of covariates affecting selection $(N_{cs}) = 2$, and total effect on selection r2=0.05

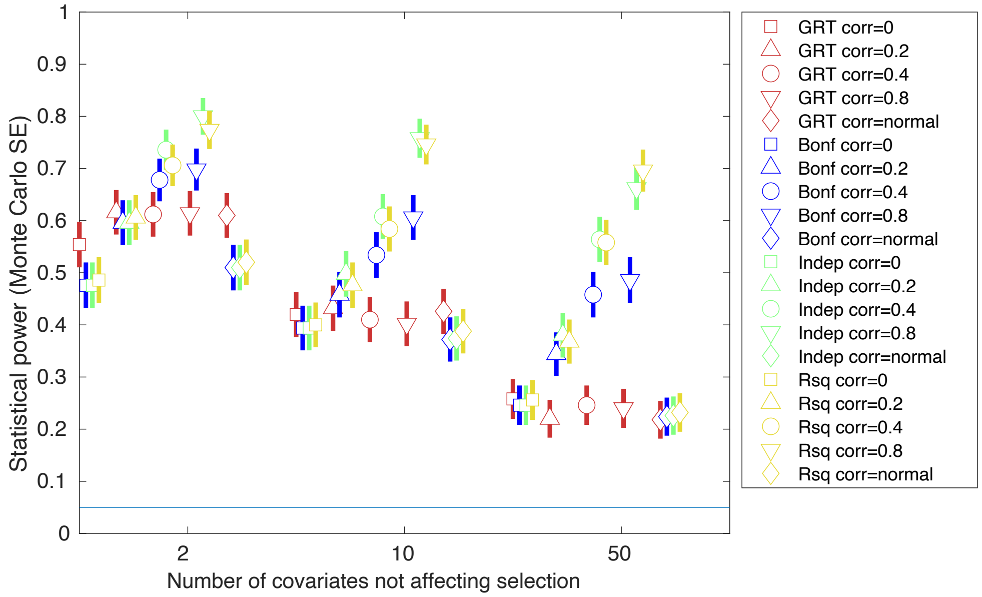

b) Horizontal pleiotropy simulation: SNP effect on each covariate R^2= 0.001

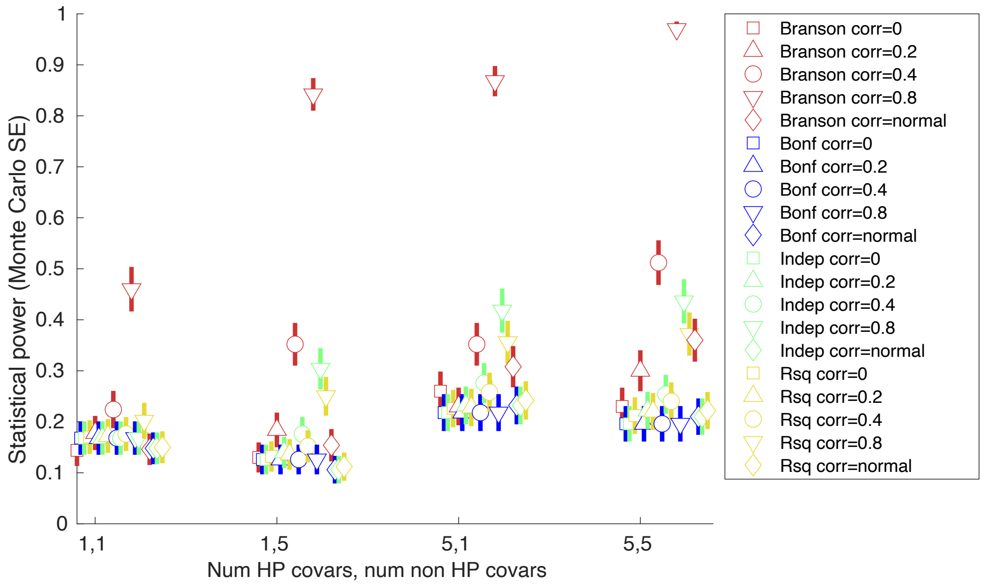

Plot (a) Is equivalent to top left plot in figure 3 of main paper. Plot (b) is equivalent to figure 4(a) in main paper.

Supplementary figure 10: Results of horizontal pleiotropy applied example

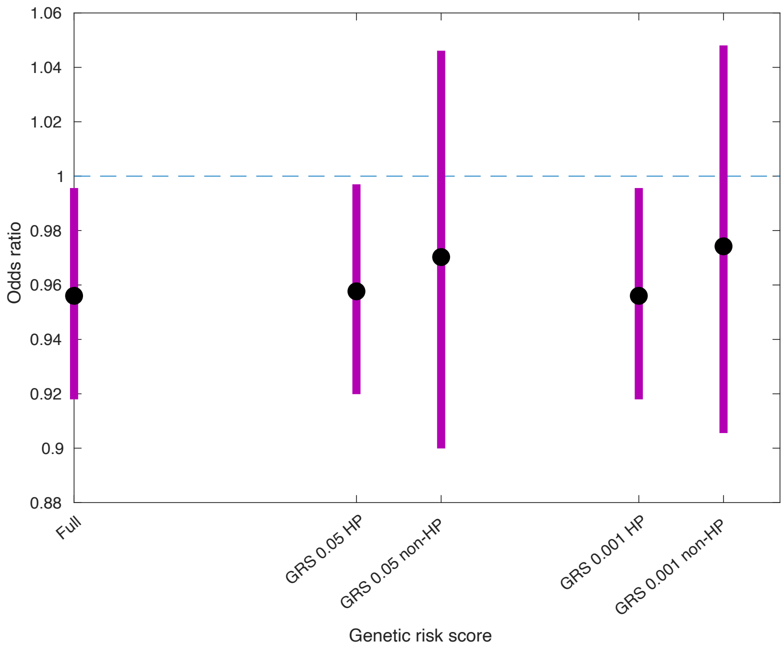

GRS: genetic risk score; HP: horizontal pleiotropy.
Full: GRS with all 58 CRP SNPs.
GRS 0.05 HP: GRS with the 51 SNPs found to be associated with the covariates set using a P=0.05 threshold.
GRS 0.05 HP: GRS with the 7 SNPs not found to be associated with the covariates set using a P=0.05 threshold.
GRS 0.001 HP: GRS with the 46 SNPs found to be associated with the covariates set using a P=0.001 threshold.
GRS 0.001 HP: GRS with the 12 SNPs not found to be associated with the covariates set using a P=0.001 threshold.

Supplementary figure 11: Relationship between number of tests and Bonferroni / independent test P value thresholds

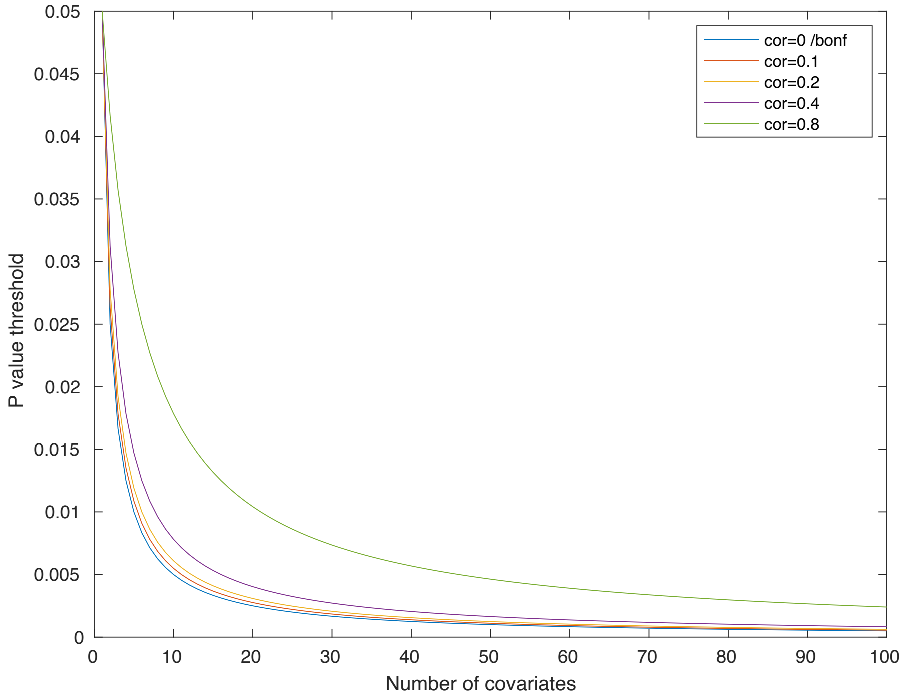

Supplementary figure 12: DAGs showing equivalent scenarios for time-varying exposures

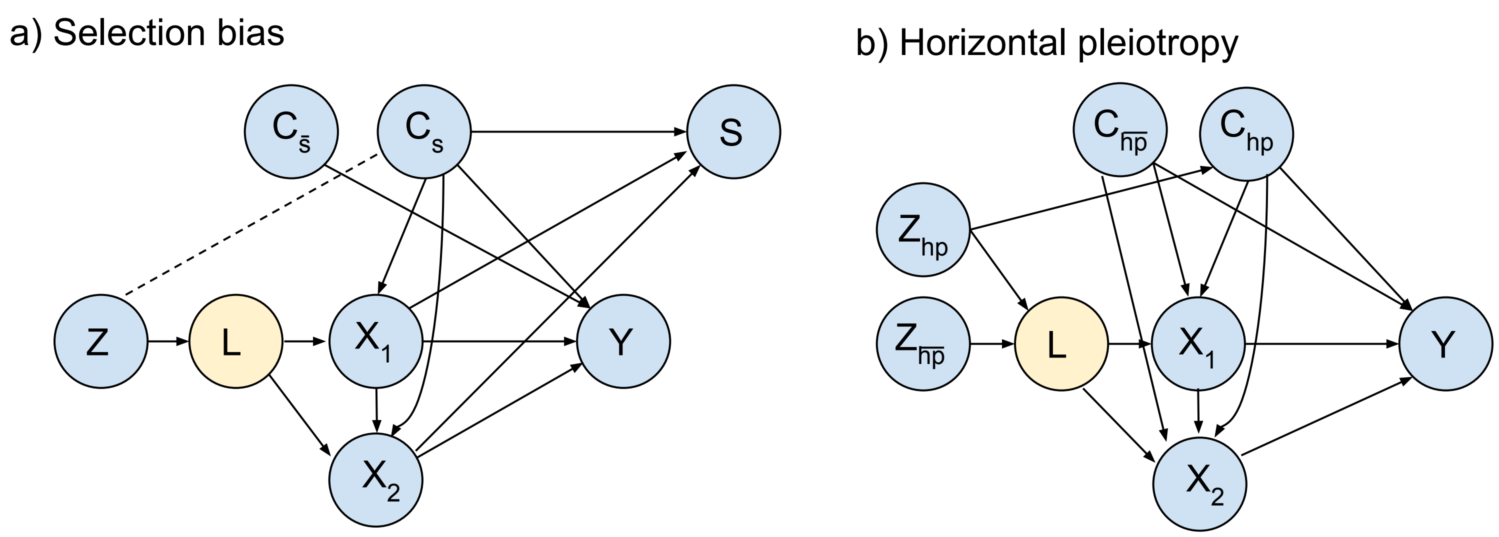

DAGs shown in Figure 2, extended to the case where the exposure X varies across the life-course. For illustration we include X at two timepoints, X_1 and X_2. When the exposure X varies across the life-course, the MR effect estimate can be interpreted as the effect on the outcome of the entire change in exposure (across the life-course) due to a change in genetic IV [3]. As we show here, in a DAG this is represented by a latent variable L that is caused by the genetic IV and causes the exposure at all timepoints [3]. In DAG (a) selection into the analytical sample (S) can be affected differently by X_1 and X_2, and testing the relationship between Z and the covariate set is looking for any selection bias due to any effect of X across the life-course. In DAG (b) the randomization test is testing for evidence of horizontal pleiotropy across the life-course. Therefore, both scenarios assessed in the main paper (assuming a time-fixed exposure) naturally extend to the case of a time-varying exposure, using the interpretation of the MR effect estimate as the effect of the underlying exposure liability [3].

Supplementary figure 13: DAGs showing scenarios for time-varying exposures with exposure-confounder feedback

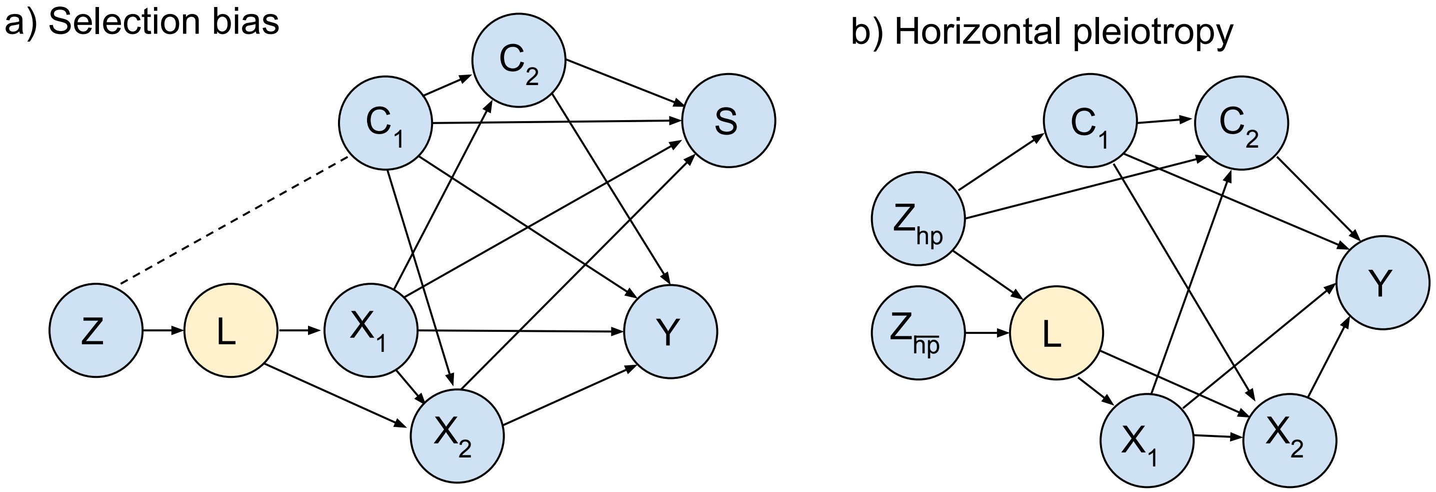

DAGs showing feedback between confounder C and exposure X, for an example with two timepoints with latent variable L. These DAGs illustrate how, when interpreting the MR effect estimate as the effect on the outcome of the entire change in exposure across the life-course using latent variable L, feedback between the exposure and confounder is not problematic because this feedback is downstream of L.

SUPPLEMENTARY TABLES

Supplementary table 1: Correlations between covariates used to assess selection bias in MR applied analysis

	**Sex**	**Age**	**Height**	**Northing**	**Easting**	**Age left continuous full-time education**	**Townsend deprivation index**
**Sex**	1	0.024	0.707	0.005	-0.006	0.029	0.013
**Age**		1	-0.107	0.022	-0.002	-0.187	-0.091
**Height**			1	-0.046	0.030	0.124	-0.053
**Northing**				1	-0.434	-0.101	-0.007
**Easting**					1	0.086	0.117
**Age left continuous full-time education**						1	-0.072
**Townsend**							1

Supplementary table 2: Correlations between covariates used in horizontal pleiotropy applied example

	P values													
**SNP**	**Smoking pack years**	**BMI**	**Weight**	**Leukocyte count**	**Albumin**	**Apol A**	**Apol B**	**Total chol**	**Glucose**	**HDL chol**	**Lipo A**	**SBP**	**DBP**	**Waist-hip ratio**
**Smoking pack years**	1	0.146	0.152	0.176	-0.078	-0.123	-0.019	-0.081	0.090	-0.150	-0.006	0.091	0.024	0.263
**BMI**		1	0.834	0.155	-0.147	-0.271	0.079	-0.049	0.160	-0.347	0.009	0.188	0.276	0.434
**Weight**			1	0.101	-0.068	-0.380	0.044	-0.116	0.142	-0.449	-0.002	0.173	0.284	0.598
**Leukocyte count**				1	-0.040	-0.099	0.015	-0.040	0.047	-0.140	-0.009	0.063	0.050	0.140
**Albumin**					1	0.115	0.110	0.145	-0.027	0.099	-0.029	0.085	0.107	0.007
**Apol A**						1	-0.027	0.319	-0.064	0.919	0.007	0.029	-0.035	-0.392
**Apol B**							1	0.887	-0.052	-0.034	0.061	0.124	0.169	0.068
**Total chol**								1	-0.093	0.335	0.054	0.105	0.131	-0.110
**Glucose**									1	-0.091	-0.005	0.110	0.037	0.160
**HDL chol**										1	0.018	-0.029	-0.087	-0.479
**Lipo A**											1	0.003	0.003	-0.007
**SBP**												1	0.698	0.224
**DBP**													1	0.241
**Waist-hip ratio**														1

Supplementary table 3: P values for individual tests of covariates with SNPs for horizontal pleiotropy applied example

	**P values**													
**SNP**	**Smoking pack years**	**BMI**	**weight**	**Leukocyte count**	**Albumin**	**Apol A**	**Apol B**	**Total chol**	**Glucose**	**HDL chol**	**Lipo A**	**SBP**	**DBP**	**Waist-hip ratio**
rs10512597	0.021	0.465	0.277	<0.001	0.007	0.005	0.865	0.276	0.869	0.009	0.749	0.889	0.166	0.641
rs1051338	0.885	0.410	0.753	<0.001	<0.001	0.025	<0.001	<0.001	0.022	0.070	0.157	0.655	0.165	0.572
rs10521222	0.855	0.778	0.200	0.807	0.005	0.328	0.095	0.368	0.668	0.460	0.560	0.671	0.711	0.197
rs10778215	0.897	<0.001	<0.001	0.625	<0.001	<0.001	0.462	0.943	<0.001	<0.001	0.128	0.882	0.084	0.012
rs10832027	0.104	<0.001	0.005	0.469	<0.001	0.241	0.707	0.657	0.920	0.001	0.084	<0.001	<0.001	<0.001
rs10838687	0.841	<0.001	0.149	0.022	0.001	<0.001	0.094	<0.001	0.001	<0.001	0.905	<0.001	<0.001	0.978
rs10925027	0.625	0.512	0.884	<0.001	<0.001	0.330	0.297	0.452	0.168	0.661	0.954	0.623	0.471	0.273
rs11108056	0.826	0.080	0.867	0.002	0.437	0.910	0.774	0.432	0.156	0.912	0.164	0.838	0.121	0.102
rs112635299	0.344	<0.001	<0.001	0.477	<0.001	0.606	0.154	0.289	0.612	0.098	0.478	<0.001	<0.001	0.005
rs1189402	0.645	0.016	0.033	0.835	0.004	0.145	0.001	0.015	0.864	0.359	0.510	0.143	0.571	0.101
rs12202641	0.094	0.174	0.002	0.354	<0.001	<0.001	<0.001	<0.001	0.252	<0.001	0.632	0.646	<0.001	0.131
rs1260326	0.806	<0.001	<0.001	<0.001	<0.001	<0.001	<0.001	<0.001	<0.001	0.124	0.237	0.255	0.394	0.346
rs12960928	0.176	<0.001	<0.001	0.191	0.021	<0.001	0.378	0.002	0.030	<0.001	0.199	0.404	0.018	<0.001
rs12995480	0.003	<0.001	<0.001	0.028	0.018	0.014	0.615	0.717	0.144	0.007	0.338	<0.001	<0.001	<0.001
rs13233571	0.559	<0.001	<0.001	<0.001	<0.001	0.448	<0.001	0.015	0.006	<0.001	0.097	0.240	0.012	0.115
rs13409371	0.895	0.369	0.461	<0.001	<0.001	0.020	0.002	<0.001	0.880	0.453	0.244	0.165	0.386	0.311
rs1441169	0.894	0.955	0.889	0.018	0.021	0.081	0.082	0.052	0.039	0.076	0.358	0.601	0.147	0.810
rs1490384	0.107	<0.001	<0.001	<0.001	0.753	0.860	<0.001	<0.001	0.003	0.027	0.914	0.720	0.015	0.006
rs1514895	0.102	<0.001	<0.001	0.215	0.082	0.103	0.261	0.058	<0.001	0.944	0.696	0.516	0.013	0.067
rs1558902	<0.001	<0.001	<0.001	<0.001	<0.001	<0.001	0.004	<0.001	<0.001	<0.001	0.336	<0.001	0.002	<0.001
rs1582763	0.192	0.009	0.117	<0.001	0.449	0.009	0.004	0.456	0.588	0.016	0.585	0.675	0.393	0.316
rs1736060	0.690	<0.001	0.002	<0.001	0.566	0.007	0.022	0.592	0.864	0.449	0.281	<0.001	<0.001	0.005
rs17658229	0.026	0.918	0.023	0.385	0.942	0.433	0.808	0.865	0.231	0.472	0.801	0.022	<0.001	0.017
rs178810	0.799	<0.001	<0.001	<0.001	0.134	0.997	0.146	0.093	0.338	0.623	0.833	0.968	0.490	0.295
rs1800961	0.111	0.893	0.316	<0.001	0.019	<0.001	<0.001	<0.001	<0.001	<0.001	0.388	0.114	0.784	0.0438
rs1805096	0.462	0.459	0.813	<0.001	<0.001	<0.001	0.593	<0.001	0.530	<0.001	0.236	0.783	0.898	0.120
rs1880241	0.433	0.212	0.696	<0.001	<0.001	0.161	0.703	0.080	0.262	0.243	0.169	<0.001	0.126	0.402
rs2064009	0.484	0.001	0.002	0.300	0.012	0.002	0.088	0.588	0.061	0.014	0.153	0.313	0.164	0.028
rs2239222	0.337	0.525	0.513	0.620	0.007	0.495	0.762	0.955	0.392	0.496	0.305	0.914	0.051	0.177
rs2293476	0.917	<0.001	<0.001	0.290	<0.001	<0.001	0.203	<0.001	0.085	<0.001	0.190	0.888	<0.001	<0.001
rs2315008	0.010	0.002	<0.001	<0.001	0.014	<0.001	0.037	0.001	0.098	<0.001	0.484	0.080	<0.001	0.120
rs2352975	0.559	<0.001	<0.001	<0.001	0.994	<0.001	0.013	<0.001	0.021	<0.001	0.256	<0.001	<0.001	<0.001
rs2710804	0.190	0.326	0.898	<0.001	<0.001	0.271	0.144	0.038	0.023	0.280	0.773	0.003	0.029	0.524
rs2794520	0.818	0.315	0.488	0.674	0.393	0.540	0.014	0.007	0.346	0.376	0.969	0.402	0.518	0.929
rs2836878	0.559	0.550	0.773	<0.001	<0.001	0.055	0.098	0.006	0.289	0.157	0.346	0.344	0.429	0.959

rs2852151	0.880	0.137	0.290	0.018	0.895	0.626	0.025	0.166	0.953	0.326	0.769	0.051	0.212	0.479
rs2891677	0.030	0.003	0.381	<0.001	0.701	0.747	0.839	0.990	0.426	0.412	0.795	0.895	0.758	0.263
rs340005	0.278	<0.001	<0.001	0.046	<0.001	<0.001	0.005	0.531	<0.001	0.021	0.755	0.006	0.022	0.844
rs4092465	0.947	0.580	0.224	0.175	0.251	0.251	0.423	0.597	0.966	0.504	0.346	0.453	0.312	0.106
rs4129267	0.586	0.932	0.539	0.223	<0.001	<0.001	0.175	<0.001	0.180	0.391	0.240	0.160	0.827	0.455
rs4246598	0.864	0.103	0.056	0.743	0.325	0.169	0.001	0.005	0.095	0.699	0.774	0.796	0.106	0.846
rs4420638	0.091	<0.001	<0.001	<0.001	0.730	<0.001	<0.001	<0.001	0.001	<0.001	0.236	0.406	0.388	<0.001
rs469772	0.125	0.207	0.280	0.640	0.141	0.009	0.001	<0.001	0.141	0.993	0.253	0.177	0.174	0.142
rs4774590	0.164	<0.001	<0.001	0.108	0.273	0.385	0.100	0.118	0.018	0.277	0.279	0.666	0.216	0.017
rs4841132	0.660	0.701	0.946	0.028	<0.001	<0.001	<0.001	<0.001	<0.001	<0.001	0.984	0.308	0.004	0.967
rs6001193	0.255	0.393	0.169	0.160	0.107	<0.001	0.125	0.751	0.671	<0.001	0.011	0.268	0.037	0.010
rs643434	0.008	0.947	0.823	<0.001	0.002	<0.001	<0.001	<0.001	<0.001	<0.001	0.004	0.637	<0.001	0.780
rs687339	0.010	<0.001	<0.001	0.113	<0.001	<0.001	<0.001	0.221	0.012	<0.001	0.007	<0.001	0.011	0.001
rs7121935	0.159	0.121	0.997	0.876	<0.001	0.033	0.497	0.628	0.122	0.486	0.423	0.638	0.028	0.263
rs7310409	0.371	0.819	0.342	0.007	0.003	<0.001	<0.001	<0.001	0.765	<0.001	0.630	0.007	0.004	0.541
rs75460349	0.848	0.007	0.569	0.021	0.276	<0.001	<0.001	<0.001	0.830	<0.001	0.473	0.019	0.015	0.454
rs7795281	0.056	<0.001	0.002	0.424	0.980	0.593	0.379	0.179	0.024	0.734	0.708	0.529	<0.001	0.067
rs9271608	0.331	0.060	0.609	<0.001	0.346	<0.001	0.003	0.168	<0.001	0.001	0.354	0.473	<0.001	<0.001
rs9284725	0.985	0.795	0.119	0.185	0.045	0.146	0.473	0.444	0.366	0.523	0.205	0.139	0.340	0.087
rs9385532	0.218	<0.001	<0.001	<0.001	0.203	0.012	<0.001	<0.001	0.635	0.011	0.803	0.071	0.363	0.008
rs9611441	0.195	0.183	0.265	0.635	0.150	0.544	<0.001	<0.001	0.166	0.646	0.351	0.072	0.445	<0.001
X17.58001690_GA_G	0.085	0.175	0.318	<0.001	<0.001	0.047	0.206	0.148	0.134	0.617	0.539	<0.001	0.006	0.550
X3.47431869_GTCT_G	0.126	0.057	0.386	<0.001	0.014	<0.001	0.384	0.144	0.252	<0.001	0.433	0.691	0.412	0.005

Supplementary table 4: Results of horizontal pleiotropy applied example – identifying horizontally pleiotropic CRP SNPs

	**P value**			
**SNP**	**Global Randomization test**	**R^2 permutation test (r^2perm)**	**Bonferroni corrected (test-Bonf) (14 tests) ***	**Independent (test-indep) (12.0 tests) ***
rs10512597	<0.001	<0.001	<0.001	<0.001
rs1051338	<0.001	0.012	<0.001	<0.001
rs10521222	**0.176**	**0.144**	**0.072**	**0.062**
rs10778215	<0.001	<0.001	<0.001	<0.001
rs10832027	<0.001	0.007	<0.001	<0.001
rs10838687	<0.001	<0.001	<0.001	<0.001
rs10925027	<0.001	<0.001	<0.001	<0.001
rs11108056	0.015	**0.108**	0.023	0.020
rs112635299	<0.001	<0.001	<0.001	<0.001
rs1189402	0.040	**0.101**	0.020	0.017
**rs**12202641	<0.001	<0.001	<0.001	<0.001
rs1260326	<0.001	<0.001	<0.001	<0.001
rs12960928	<0.001	<0.001	<0.001	<0.001
rs12995480	<0.001	<0.001	<0.001	<0.001
rs13233571	<0.001	<0.001	<0.001	<0.001
rs13409371	<0.001	<0.001	<0.001	<0.001
rs1441169	0.006	**0.321**	**0.256**	**0.219**
rs1490384	<0.001	<0.001	<0.001	<0.001
rs1514895	<0.001	<0.001	<0.001	<0.001
rs1558902	<0.001	<0.001	<0.001	<0.001
rs1582763	0.006	0.032	<0.001	<0.001
rs1736060	<0.001	0.005	<0.001	<0.001
rs17658229	<0.001	0.012	<0.001	<0.001
rs178810	<0.001	0.009	<0.001	<0.001
rs1800961	<0.001	<0.001	<0.001	<0.001
rs1805096	<0.001	<0.001	<0.001	<0.001
rs1880241	<0.001	<0.001	<0.001	<0.001
rs2064009	<0.001	**0.076**	0.009	0.008
rs2239222	**0.161**	**0.171**	**0.096**	**0.083**
rs2293476	<0.001	<0.001	<0.001	<0.001
rs2315008	<0.001	<0.001	<0.001	<0.001

rs2352975	<0.001	<0.001	<0.001	<0.001
rs2710804	<0.001	<0.001	<0.001	<0.001
rs2794520	**0.847**	**0.212**	**0.097**	**0.083**
rs2836878	<0.001	<0.001	<0.001	<0.001
rs2852151	**0.551**	**0.360**	**0.250**	**0.214**
rs2891677	<0.001	0.002	<0.001	<0.001
rs340005	<0.001	<0.001	<0.001	<0.001
rs4092465	**0.311**	**0.827**	**1.000**	**1.000**
rs4129267	<0.001	<0.001	<0.001	<0.001
rs4246598	0.029	**0.095**	0.021	0.018
rs4420638	<0.001	<0.001	<0.001	<0.001
rs469772	<0.001	0.017	<0.001	<0.001
rs4774590	**0.055**	**0.061**	0.005	0.004
rs4841132	<0.001	<0.001	<0.001	<0.001
rs6001193	<0.001	0.001	<0.001	<0.001
rs643434	<0.001	<0.001	<0.001	<0.001
rs687339	<0.001	<0.001	<0.001	<0.001
rs7121935	<0.001	0.002	<0.001	<0.001
rs7310409	<0.001	<0.001	<0.001	<0.001
rs75460349	<0.001	<0.001	<0.001	<0.001
rs7795281	<0.001	<0.001	<0.001	<0.001
rs9271608	<0.001	<0.001	<0.001	<0.001
rs9284725	**0.153**	**0.500**	**0.631**	**0.541**
rs9385532	<0.001	<0.001	<0.001	<0.001
rs9611441	<0.001	0.003	<0.001	<0.001
X17.58001690_GA_G	<0.001	<0.001	<0.001	<0.001
X3.47431869_GTCT_G	<0.001	<0.001	<0.001	<0.001

P values shown in bold are those >0.05, such that the SNP is not identified as being horizontally pleiotropic.

REFERENCES

1. Swanson JM. The UK Biobank and selection bias. Lancet. 2012;380:110.
2. Morris TP, White IR, Crowther MJ. Using simulation studies to evaluate statistical methods. Stat Med. 2019;38:2074–102.
3. Morris TT, Heron J, Sanderson ECM, Davey Smith G, Didelez V, Tilling K. Interpretation of Mendelian randomization using a single measure of an exposure that varies over time. Int J Epidemiol. 2022;51:1899–909.
